# A proposal for the assessment of replication of effects in single‐case experimental designs

**DOI:** 10.1002/jaba.923

**Published:** 2022-04-25

**Authors:** Rumen Manolov, René Tanious, Belén Fernández‐Castilla

**Affiliations:** ^1^ Department of Social Psychology and Quantitative Psychology University of Barcelona; ^2^ Psychology and Educational Sciences, Methodology of Educational Sciences Research Group KU Leuven – University of Leuven Leuven Belgium

**Keywords:** single‐case experimental designs, replication, visual analysis, Brinley plot, expert judgment

## Abstract

In science in general and in the context of single‐case experimental designs, replication of the effects of the intervention within and/or across participants or experiments is crucial for establishing causality and for assessing the generality of the intervention effect. Specific developments and proposals for assessing whether an effect has been replicated or not (or to what extent) are scarce, in the general context of behavioral sciences, and practically null in the single‐case experimental designs context. We propose an extension of the modified Brinley plot for assessing how many of the effects replicate. To make this assessment possible, a definition of replication is suggested, on the basis of expert judgment, rather than on statistical criteria. The definition of replication and its graphical representation are justified, presenting their strengths and limitations, and illustrated with real data. A user‐friendly software is made available for obtaining automatically the graphical representation.

Replication in science in general and in single‐case experimental designs (SCEDs) in particular is crucial, as widely acknowledged in the main SCED textbooks (e.g., Kazdin, 2020; Kennedy, 2005; Ledford & Gast, 2018; Morley, 2018; Riley‐Tillman et al., 2020; Sidman, 1960; Tate & Perdices, 2019; U.S. Department of Education, 2020) and in the What Works Clearinghouse standards (Kratochwill et al., 2013; U.S. Department of Education, 2020). Replication has also been recently emphasized in journal articles, both in the SCED context (e.g., Hantula, 2019; Kazdin, 2021; Lanovaz et al., 2019; Nikles et al., 2021; Tincani & Travers, 2019; Walker & Carr, 2021) and in other research contexts related to the behavioral sciences (Dixon & Glover, 2020; Hedges, 2019; Hillary & Medaglia, 2020). Specifically, relying on the principles of SCED research instead of statistical significance and the nomothetic approach^1^ has been mentioned among the possible ways to deal with the replication crisis (Hillary & Medaglia, 2020; Iversen, 2021; Tincani & Travers, 2019), although there are many possible reasons for this crisis, in relation to the data‐analytical decisions that researchers continuously make (Laraway et al., 2019).

Following Sidman's (1960) classification, direct replication is designed to identify the reliability of a finding, whereas systematic (sometimes also called conceptual) replication is designed to identify its generality (Tincani & Travers, 2019; Walker & Carr, 2021). Direct replication usually takes place within the same study, and it can be either an intrasubject replication (e.g., an ABAB or an alternating‐treatments design) or an intersubject replication, as in a multiple‐baseline design (Riley‐Tillman et al., 2020). Systematic replication entails introducing planned modifications of the original study by altering features of the setting, the behavior, the participant(s), or some component(s) of the intervention, as well as the research team carrying out the study (Horner et al., 2005; Tate & Perdices, 2019). In that sense, systematic replication usually takes place across studies (Kennedy, 2005).

Thus, replication is relevant both for internal and external validity. For generalization or external validity, several pieces of information regarding the participant, the intervention, and the setting are relevant when assessing the degree to which an effect observed in one study or in a set of studies can be expected to generalize beyond the existing studies (Hitchcock et al., 2015; Maggin, 2015). Further, several replications are necessary for identifying when, where, and with whom an intervention is and is not likely to be effective (Walker & Carr, 2021).

As an additional use, replication is important to help resolve the uncertainty that can arise if different visual criteria and different statistical analytical options lead to different conclusions in the context of a single study (Kazdin, 2020). That is, if the study is replicated and a positive effect of the intervention is observed repeatedly, the degree of uncertainty will be reduced. Replication is not only relevant in applied domains (e.g., replication of intervention effects), but also when carrying out methodological studies on SCED data analytical procedures (e.g., Bishara et al., 2021; Falligant et al., 2020). Thus, it is important to have an objective way of defining whether the results of different replications agree or not.

## Developments Needed for Assessing Replications

Given the importance of replication, it is relevant to consider how it has been suggested to be assessed both in the SCED field and in the wider scientific context. An initial data analytical approach to replication has consisted of questioning the usefulness of *p*‐values (Cumming, 2008; Sanabria & Killeen, 2007). More recently, Schauer et al. (2021) stated that, “Greater effort should be devoted to ensuring that any proposed analysis method aligns with clear and justifiable definitions of replication” (p. 18). Thus, it is important to first define replication in a manner that is consistent with the research aims. Another recommendation, in relation to replication was made by Maggin (2015), who suggested that increased access to data visualization tools might serve an important purpose in effectively communicating results across several replication attempts. He pointed at the need to actively develop and refine methods for coding, organizing, and presenting the information drawn from a series of replication attempts. In the current text, we propose a definition of direct replication and a graphical way of assessing the degree to which basic effects (i.e., A‐B comparisons; Horner & Odom, 2014) are replicated in the SCED context.

Previous proposals for the assessment of replication have taken place outside of the SCED context and have been based on inferential statistics, including more complex technical details and assumptions to determine whether required sampling distributions are adequate. On the one hand, Killeen (2005) proposed the probability of replication (labeled *p*_rep_) as an alternative to the typically used *p*‐values (i.e., the probability of observing such an extreme result as the one observed, or a more extreme one, in case the null hypothesis is true). The probability of replication (*p*_rep_) quantifies, after a positive effect has been observed, the probability that another positive effect would be obtained, where the term “positive” is related to a pre‐established minimum effect size. Further details were provided by Sanabria and Killeen (2007), also in the context of examples with comparing groups. On the other hand, the homogeneity test (Q‐statistic referred to a chi‐square distribution) from the meta‐analysis context has been suggested (Hedges, 2019; Hedges & Schauer, 2019b). When the Q‐statistic is used, it is necessary to define a negligible heterogeneity that would not be considered as evidence against replication. Furthermore, it is necessary to consider several aspects: (a) whether the burden of proof lies with replication or with failure to replicate (i.e., how the null hypothesis is structured and whether the evidence for replication would require accepting or rejecting the null hypothesis); (b) when replication is defined as exact or approximate; and (c) whether the studies are conceived as a fixed set or a random sample from a population (Hedges & Schauer, 2019b). After reviewing several options, “the major conclusion about testing hypotheses about replication is that different tests are possible and the choice among them is not automatic, but a principled analytic decision that requires some care” (Hedges, 2019, p. 11). Although none of these options is a direct antecedent for the proposal made here, they do refer to two important considerations including: (a) establishing a priori how much of an effect is desired, and (b) establishing a priori how much variability in effects is acceptable, both of which are applicable to the current proposal.

## Aim and Organization of the Text

The aim of the current text is to propose a simple visual descriptive tool for assessing the degree to which an effect has been replicated within a study or across studies. This tool requires expert judgment for its definition, rather than an arbitrarily pre‐established numerical cut‐off. In that sense, the aim was to avoid statistical inferential procedures and the assumptions they require. Consequently, the current proposal circumvents null hypothesis testing which may not be of interest for a behavior analyst using a SCED (e.g., Hartgerink et al., 2017). Moreover, it also does not entail potentially problematic comparisons of *p*‐values and the need to be concerned with statistical power (Schauer et al., 2021). Finally, it does not require using more sophisticated options such as Bayesian analysis (Etz & Vandekerckhove, 2016), which can be harder to learn (Natesan, 2019) and are not likely to be included in courses for applied researchers (Wolfe & McCammon, 2022). Thus, the application of the proposal does not require applied researchers to learn complex statistical analyses or software (Brown et al., 2019).

In pursuit of this aim, the text is organized as follows. First, a graphical representation called the modified Brinley plot (Blampied, 2017) is presented, with its main features, strengths, and limitations. Second, the proposal is based on the modified Brinley plot, including its methodological framework, rationale, and examples. Limitations and challenges related to the proposal are also identified. Third, we explain, step‐by‐step, how software developed for implementing the proposal can be used. Finally, a discussion of the implications of the proposal is presented.

## The Modified Brinley Plot

### Main Features

A modified Brinley plot (Blampied, 2017) allows representation of an effect (i.e., a comparison between an A condition, such as a baseline, and a B condition, such as an active intervention) as a dot, whose coordinates are defined by the Phase A mean and the Phase B mean. An identity line (diagonal with intercept = 0, slope = 1) is included to represent the lack of difference between means. The dots above the line indicate that the Phase B means are greater than the corresponding Phase A means, whereas dots below the line indicate that Phase B means are smaller than the corresponding Phase A means. On such a plot, several effects can be represented, within and across participants. Raw data are not represented, but only summaries such as within‐phase means. It is also possible to include additional visual aids for representing the desired magnitude of intervention effectiveness and a cut‐off point representing the normative range of the target behavior (Blampied, 2017). The modified Brinley plot is similar to the L'Abbé plot (L'Abbé et al., 1987) used in meta‐analysis for visually assessing the consistency of effects across studies (Anzures‐Cabrera & Higgins, 2010).

### Usefulness for Representing Effects Within and Across Studies

#### Within‐Study Example: Multiple Baseline Design

**Figure 1:**
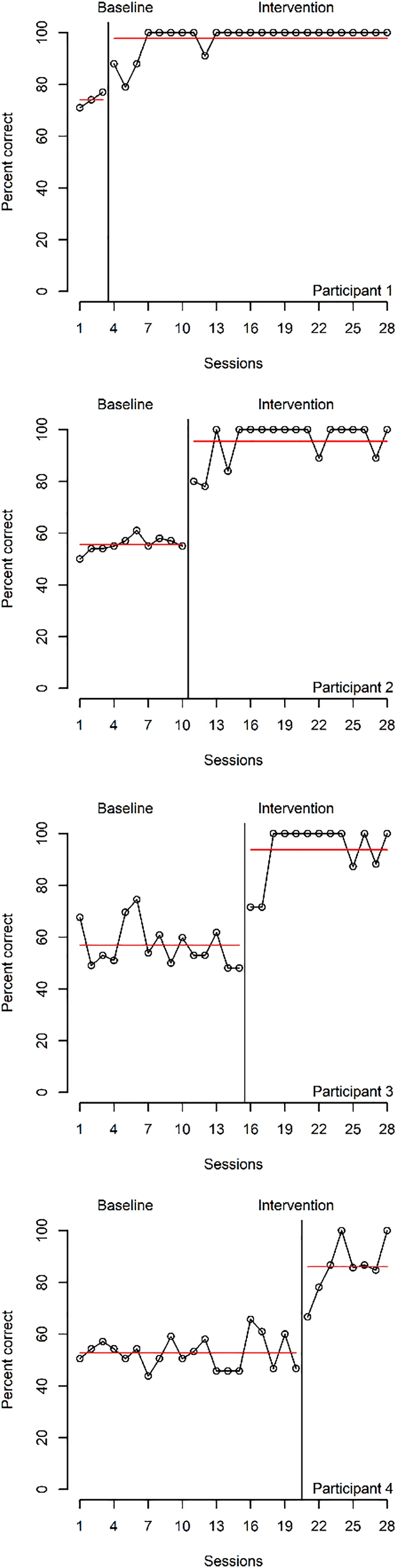
Raw Data for the Percentage of Correctly Filed Items Across Four Children, Gathered by Dorminy et al. (2009)

**Figure 2:**
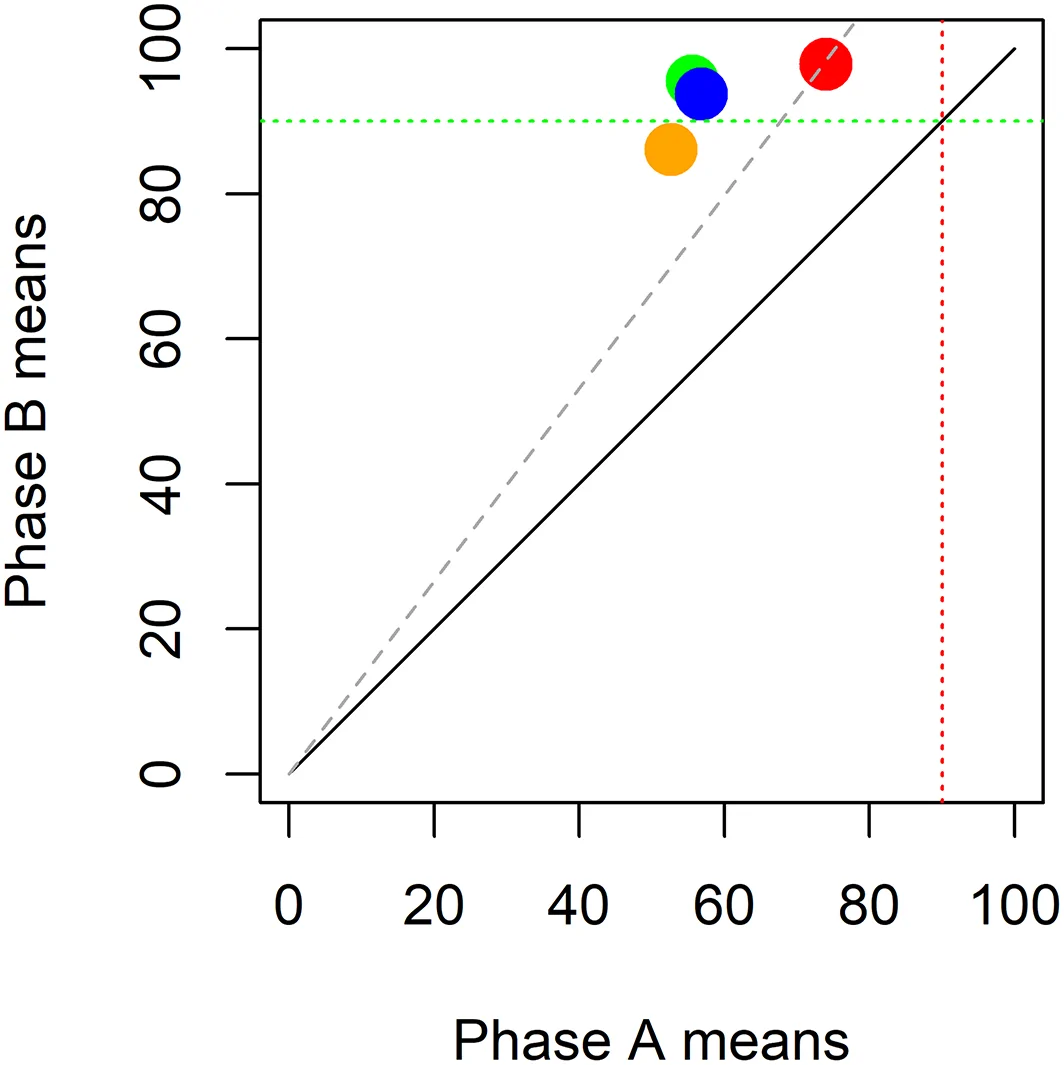
Modified Brinley Plot for Dorminy et al. (2009) *Note*. Each dot represents an A‐B comparison, with the x‐axis coordinate defined by the baseline mean and the y‐axis coordinate defined by the intervention phase mean. The green horizontal dotted line represents the desired postintervention level, whereas the red vertical dotted represents whether this level was already present during the baseline phase. The grey dashed diagonal line represents the desired amount of change from the baseline level.

Dorminy et al. (2009) used a multiple‐baseline design across four participants and across two behaviors to teach organizational skills to children diagnosed with autism spectrum disorder and Asperger's syndrome. Figure 1 shows the time‐series line graph for the percentage of correctly filed items, whereas Figure 2 shows the modified Brinley plot for the same data. The relatively stable baseline and intervention levels suggest that using means, as in the modified Brinley plot, is reasonable. We did not depict the second behavior (number of seconds it took students to locate specific items) in the same modified Brinley plot, as it was expressed in different measurement units.

In Figure 2, each dot is an A‐B comparison, and the dots have four different colors, as they belong to four different participants. The y‐axis represents the mean of the measurements in Phase B, whereas the x‐axis represents the mean of the measurements in Phase A. All dots are above the solid diagonal identity line, indicating that the intervention phase average level (Phase B, y‐axis) is higher than the Phase A average level (Phase A, x‐axis) for all participants. Additionally, we added a green horizontal line representing a supposed desired postintervention level of 90. Three of the Phase B means are above this line. There is also a red vertical line which indicates whether any Phase A means were already above 90 even before the intervention (i.e., to the right of the red vertical line), which is not the case for these data. Finally, we added a grey dashed diagonal line representing a supposed desired improvement of 33% over the Phase A level. The desired amount of improvement, specified as a percentage increase, entails that for higher preintervention (Phase A) values, the amount of change required after the intervention (Phase B) is larger. For a Phase A mean of 55 (which is similar to the Phase A mean for three of the participants), this would entail requiring an intervention mean of at least 55+55×0.33=55×1.33=73.15. All three participants with a Phase A mean of approximately 55 had higher Phase B means than 73.15. For the participant with a Phase A mean of 74, the required level was 98.42 and it was not achieved (the rightmost dot is below the grey dashed line).

#### Within‐Study Example: (Replicated) Alternating Treatments Design

**Figure 3:**
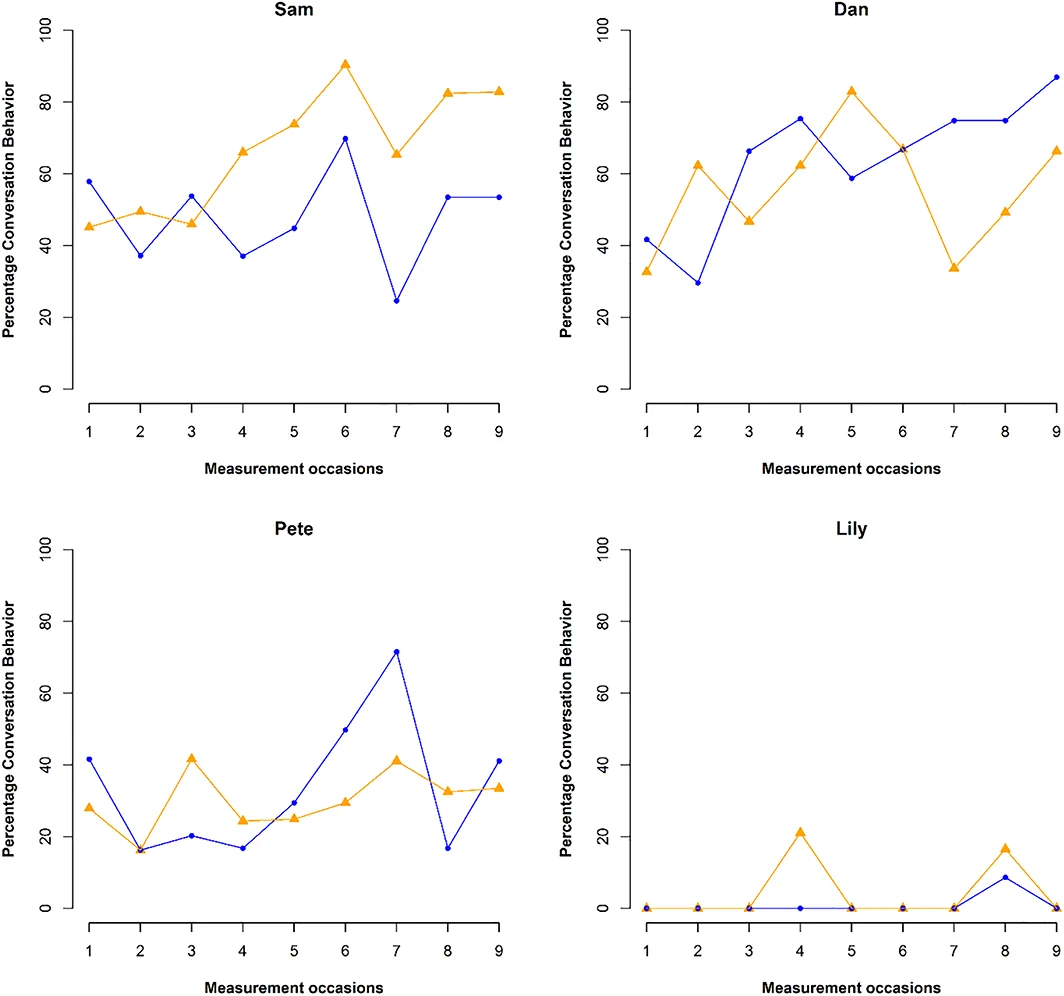
Raw Data for the Percentage of Conversation Behavior, Gathered by Thirumanickam et al. (2018)

Thirumanickam et al. (2018) performed a comparison between video modeling and video self‐modeling interventions to develop conversational behaviors with four adolescents with autism spectrum disorder who used augmentative and alternative communication. The data for the comparison phase in which the two interventions were alternated are presented in Figure 3. It should be noted that the graph reproduces the one by Thirumanickam et al. (2018), not including a specific order for the two conditions in each session (i.e., for each measurement occasion there is one measure that belongs to each condition). This makes the direct vertical comparison between measurements belonging to different conditions easier.^2^ The raw data do not show a clear superiority of either condition for all four participants. Although the later measurement occasions for Sam and Dan suggested clearer differentiation, the condition that is superior in these final measurement occasions is not the same for both participants.

**Figure 4:**
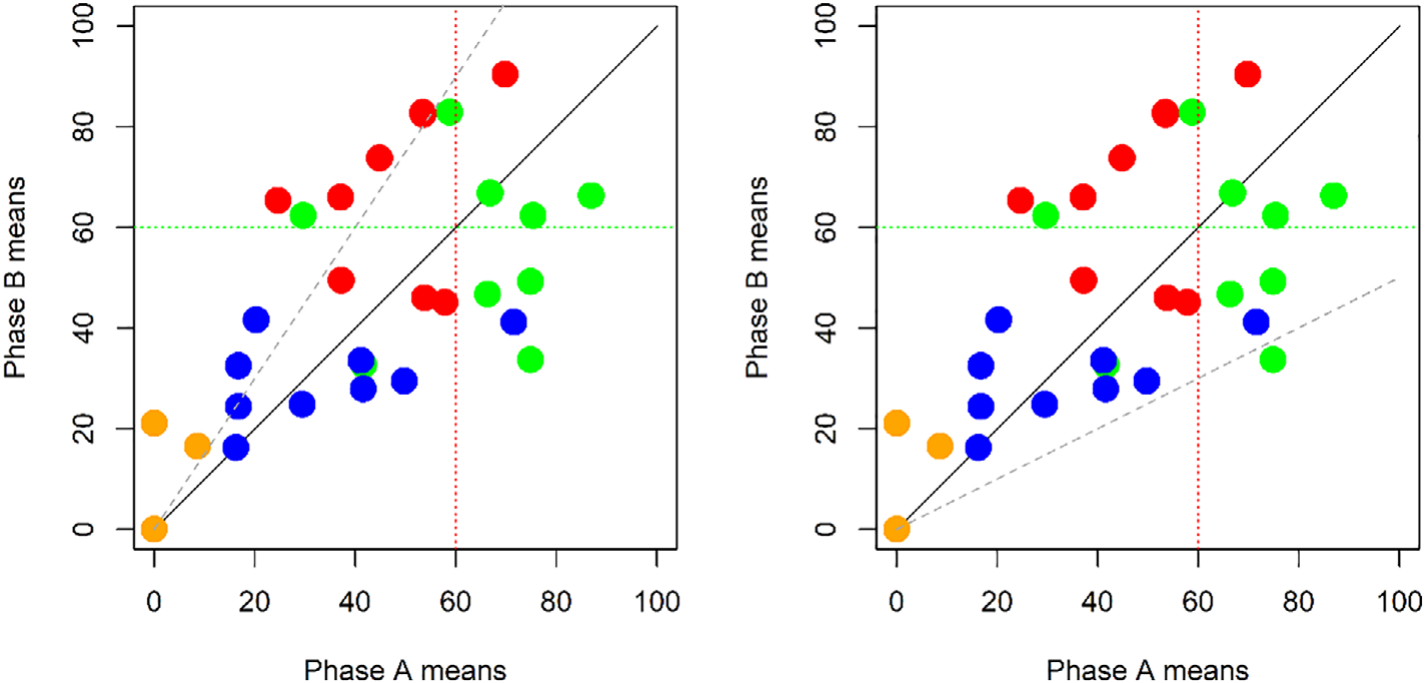
Modified Brinley Plot for Thirumanickam et al. (2018) *Note*. Each dot represents an A‐B comparison, with the x‐axis coordinate defined by the baseline mean and the y‐axis coordinate defined by the intervention phase mean. Dots of the same color belong to the same participant. The green horizontal dotted line represents the desired postintervention level whereas the red vertical dotted line represents whether this level was already present during the baseline phase. The grey dashed diagonal line represents the desired amount of change from the baseline level: left panel ‐ expected increase; right panel ‐ expected reduction.

The modified Brinley plot is represented in Figure 4, with dots of the same color corresponding to the same participant. There are nine dots per participant, as there are that many comparisons between the two conditions (i.e., there are nine alternations of the A and B conditions). The impression of a lack of superiority of one of the conditions is also reflected here, considering where the dots are located with respect to the solid diagonal identity line. Specifically, for Participant 1 (marked in red; with most dots above the solid diagonal line), condition B is superior, for Participant 2 (marked in green; with most dots below the solid diagonal line), condition A is superior. For Participant 3 (marked in blue; with dots scattered around the solid diagonal line) there is no clear superiority of either condition. For Participant 4 (marked in yellow; with dots in the lower left corner), there are some comparisons with superiority of condition B and some overlapped dots with the same values for both conditions (i.e., dots on the solid diagonal line). For illustrative purposes, the current authors added a horizontal green dotted line marking a supposed desired postintervention level of 60. Given that it is not directly clear which condition should be superior, we also added a dashed grey diagonal line representing a supposed desired increase of 50% (left graph of Figure 4: video self‐modeling superior to video modeling) or decrease of 50% (right graph of Figure 4: video modeling superior to video self‐modeling). Few dots are above the grey dashed diagonal line or above the green horizontal line and to the left of the red vertical line in the left graph of Figure 4a, suggesting that any potential superiority of condition B (video self‐modeling) is not a replicated effect. Similarly, only one dot is below the grey dashed diagonal line, below the green horizontal line and to the right of the red vertical line, suggesting very little evidence of any potential superiority of condition A (video modeling). Therefore, there is no clear evidence about the superiority of either condition.

The application of the modified Brinley plot is made easier when only two conditions are being compared. Therefore, in case the researchers are willing to compare two interventions and include a nonintervention condition (e.g., Skinner et al., 2021), there would be a need for a separated modified Brinley plot for each comparison between pairs of conditions. The use of the modified Brinley plot for alternating treatment designs is also more straightforward when there is the same number of measurements per condition, as is the case for block randomization (Manolov & Tanious, 2022; Onghena & Edgington, 2005).

#### Across‐Studies Example: Replicated Reversal Design

**Figure 5:**
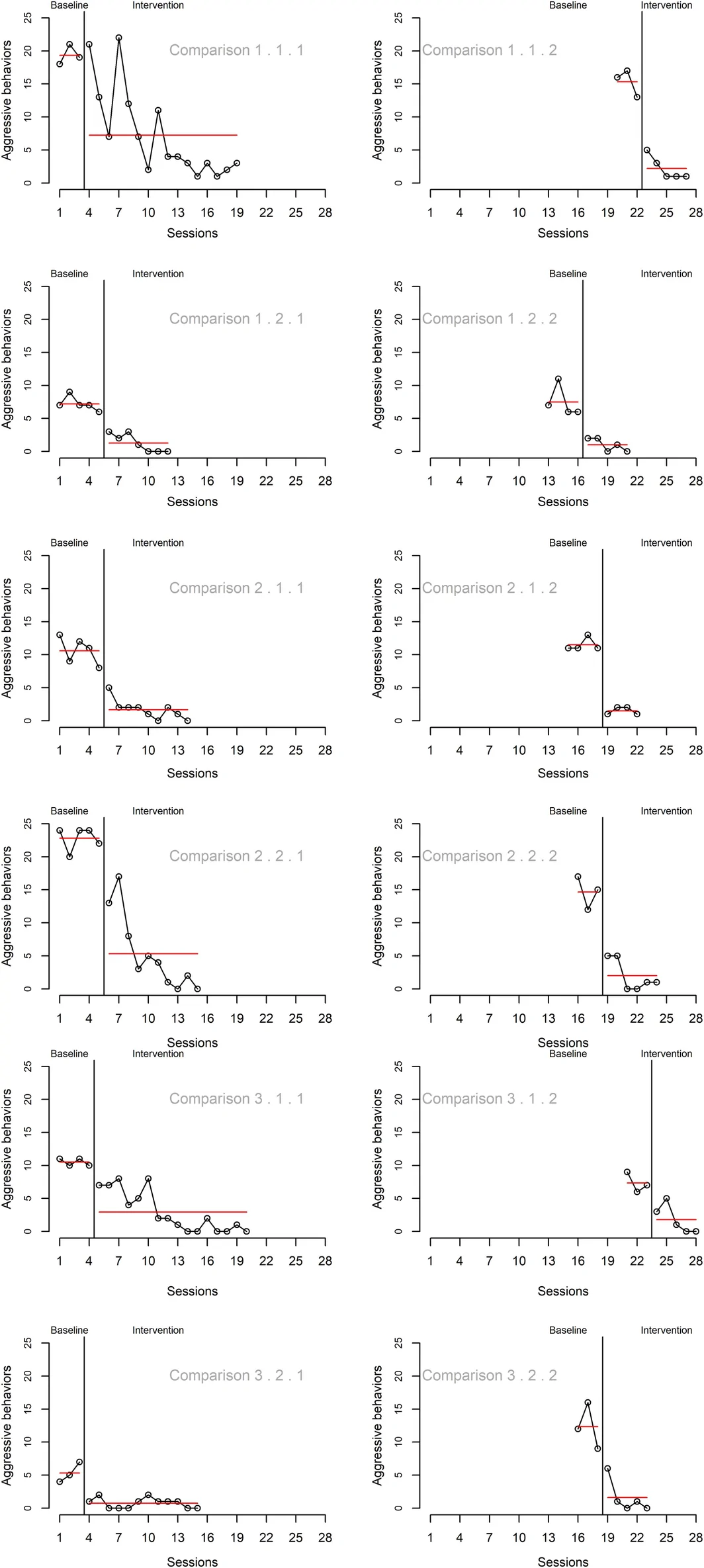
Time‐series Line Graph for Feeney and Ylvisaker (2003, 2006, 2008)

**Figure 6:**
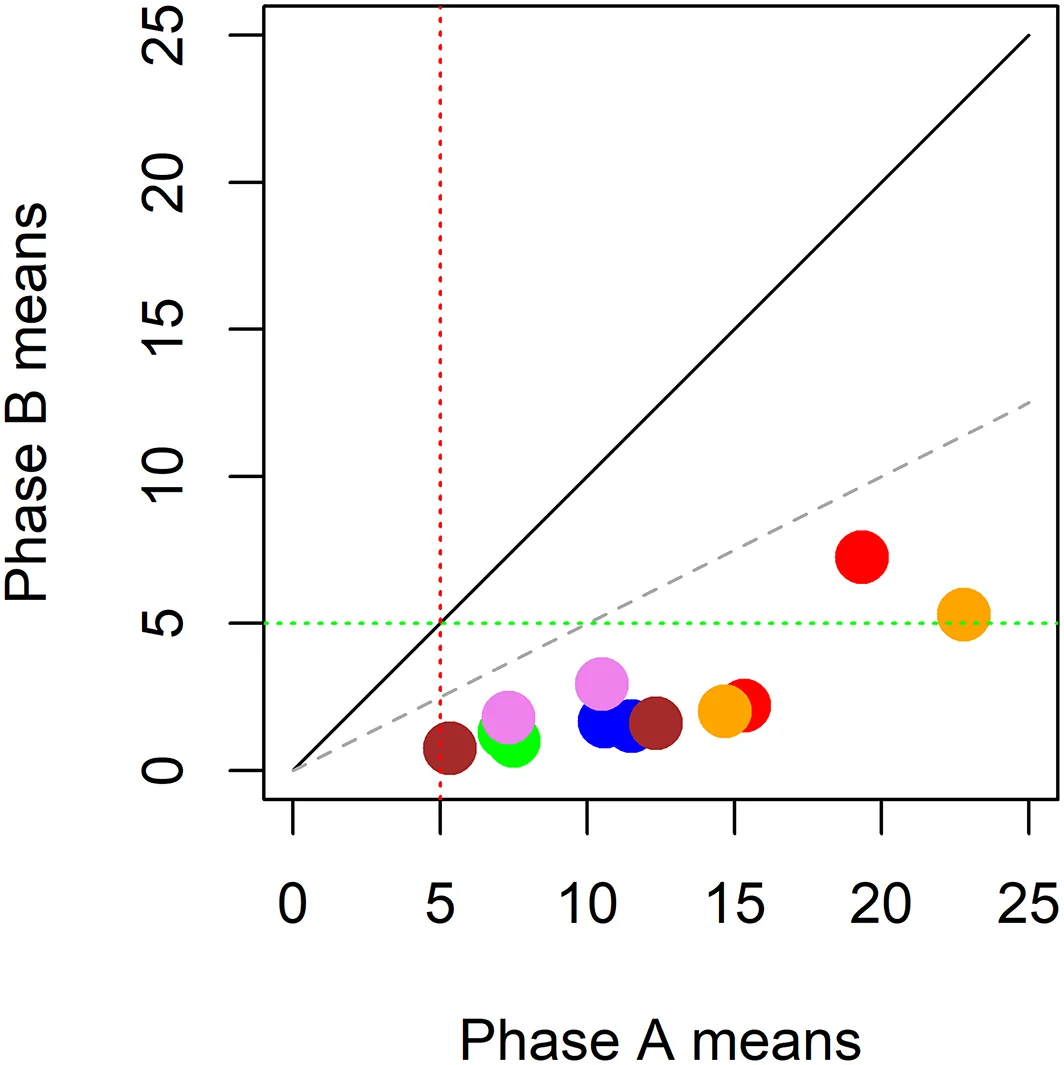
Modified Brinley Plot for Feeney and Ylvisaker (2003, 2006, 2008) *Note*. Each dot represents an A‐B comparison, with the x‐axis coordinate defined by the baseline mean and the y‐axis coordinate defined by the intervention phase mean. Dots of the same color belong to the same participant. The green horizontal dotted line represents the desired postintervention level, whereas the red vertical dotted represents whether this level was already present during the baseline phase. The grey dashed diagonal line represents the desired amount of change from the baseline level.

Feeney and Ylvisaker (2003, 2006, 2008) carried out a series of studies using context‐sensitive cognitive‐behavioral supports to reduce aggressive behaviors in young children with traumatic brain injury. In each of three studies, they used an ABAB design, replicated across two participants. The raw data for the three studies are represented in Figure 5, whereas the modified Brinley plot is represented in Figure 6. Thus, Figure 6 contains data from three studies, two participants per study, and two effects (A‐B comparisons) per participant, given that an ABAB design was followed for each participant. The modified Brinley plot entails omitting the B1‐A2 comparison that is possible in an ABAB design (i.e., performing only the A1‐B1 and A2‐B2 comparisons, which agrees with previous suggestions from the SCED context (e.g., Parker & Vannest, 2012; Tanious et al., 2020). Each dot represents an effect and thus there are 3×2×2=12 dots. There are two dots of the same color, and they belong to the same participant.

We represented, via a dotted horizontal green line, a supposed desired postintervention level of a maximum of five aggressive behaviors. This is met by all Phase B means, except for two. We also represented, via a grey dashed line, a supposed desired amount of change consisting of a reduction of 50% of the Phase A level. All comparisons meet this criterion.

### Advantages of the Use of the Modified Brinley Plot

First, we refer to the importance of graphical representations and visual analysis in general. Visual inspection has a long history in the SCED context (e.g., Miller, 1985; Parker et al., 2006) and is considered important and necessary even with the current abundance of statistical techniques (DeRosa et al., 2021; Ferron et al., 2017; Kipfmiller et al., 2019; Ledford, Barton, Severini, & Zimmerman, 2019; Maggin et al., 2018; Ninci, 2019; Wolfe et al., 2019). This is consistent with the training received by certain professionals (Wolfe & McCammon, 2022), with applied researchers' priorities when analyzing data (Byiers et al., 2021), and within actual practice (Dowdy et al., 2021). Moreover, visually inspecting data has been emphasized as indispensable when performing classical statistical analyses outside the SCED context (Fife et al., 2021). Extensions of existing methods for displaying information visually have recently been proposed both in the SCED context (Snodgrass et al., 2022) and in a broader research context (Fernández‐Castilla et al., 2020).

Second, in terms of the advantages of the modified Brinley plot in relation to the common time‐series line graphs, several aspects need to be addressed. First, the agreement between visual analysts inspecting time‐series plots has been found to be insufficient (see Ninci et al., 2015, for a meta‐analysis, and also Bishara et al., 2021; Tarlow et al., 2021). Second, the modified Brinley plot is not affected by graphical features such as the ratio between x‐axis and the y‐axis (*x*:*y* ratio; Kubina et al., 2017), given that it is square by definition. Similarly, if the aim was to merely check whether there is an improvement for all A‐B comparisons, this would be visually evident because of the diagonal line representing lack of change, regardless of the *x*:*y* ratio and the data points per *x*:*y* ratio. A great variety in these two ratios has been found in time‐series line graphs, leading to potential distortions when performing visual analysis (Kubina et al., 2017; Ledford, Barton, Severini, Zimmerman, & Pokorski, 2019; Peltier, McKenna, et al., 2022; Peltier et al., 2021; Peltier, Muharib, et al., 2022). Third, the confounding between slope and scale (Kinney, 2022) is also not likely for modified Brinley plots, given that they are square. Finally, the modified Brinley plot is efficient in that it makes possible representing the results (e.g., within‐phase means and mean differences) for several comparisons within participants and across participants on the same plot, as was illustrated previously in the text. Moreover, visual aids such as the solid diagonal line (indicating which condition is associated with better results), the green horizontal dotted line (indicating if a desired postintervention level has been achieved), and the grey dashed diagonal line (indicating if the amount of difference between conditions is sufficiently large) allow for a fast evaluation of multiple aspects.

### Limitations Using the Modified Brinley Plot

#### Loss of Information About Time

Raw measurements are not represented on the modified Brinley plot. In contrast, the time‐series line graph allows for the representation of all raw measurements in a temporal order. For instance, the fact that differentiation is achieved for later measurement occasions for two participants in the Thirumanickam et al. (2018) study, as per Figure 3, is not reflected in the corresponding modified Brinley plot (Figure 4). However, it should be noted that raw measurements are primarily important for formative analysis (Barton et al., 2016; Fahmie & Hanley, 2008; Ledford, Barton, Severini, & Zimmerman, 2019) and for studying the process and relevant mediators of the intervention effect (Caneiro et al., 2019; Hayes et al., 2019). In contrast, the extent to which effects are replicated, which is the focus of the current text, is mainly a summative analysis once the data collection is completed.

#### Loss of Information About Variability

A mean does not directly represent the number of measurements from which it is computed, and thus certain effects (mean differences) may be based on an insufficient amount of data, as per current standards (e.g., Tate et al., 2013; U.S. Department of Education, 2020). For instance, in the reversal designs used by Feeney and Ylvisaker (2003, 2006, 2008) some of the initial A‐B comparisons were based on more data, as seen in Figure 5. To address this first issue, it is possible to make the dots on the modified Brinley plot proportional to the number of measurements (Manolov et al., 2021).

However, the mean may not be a good representation of the data (Parker et al., 2011). To address this second issue, it is possible to represent on the modified Brinley plot, the degree to which the mean represents the data via horizontal and vertical lines, denoting the variability around the mean line in the Phase A and Phase B, respectively (Manolov et al., 2021). Thus, these lines can be understood as error bars and this variability is quantified via the mean absolute error (Hyndman & Koehler, 2006; Tanious et al., 2020). For instance, for the Feeney and Ylvisaker (2003, 2006, 2008) data, for Cases 3 and 6 from Figure 5, the mean does not represent the data well enough. In contrast, the means seem to be good representations of the Dorminy et al. (2009) data and a change in level seems to represent the kind of effect observed (Figure 1).

**Figure 7:**
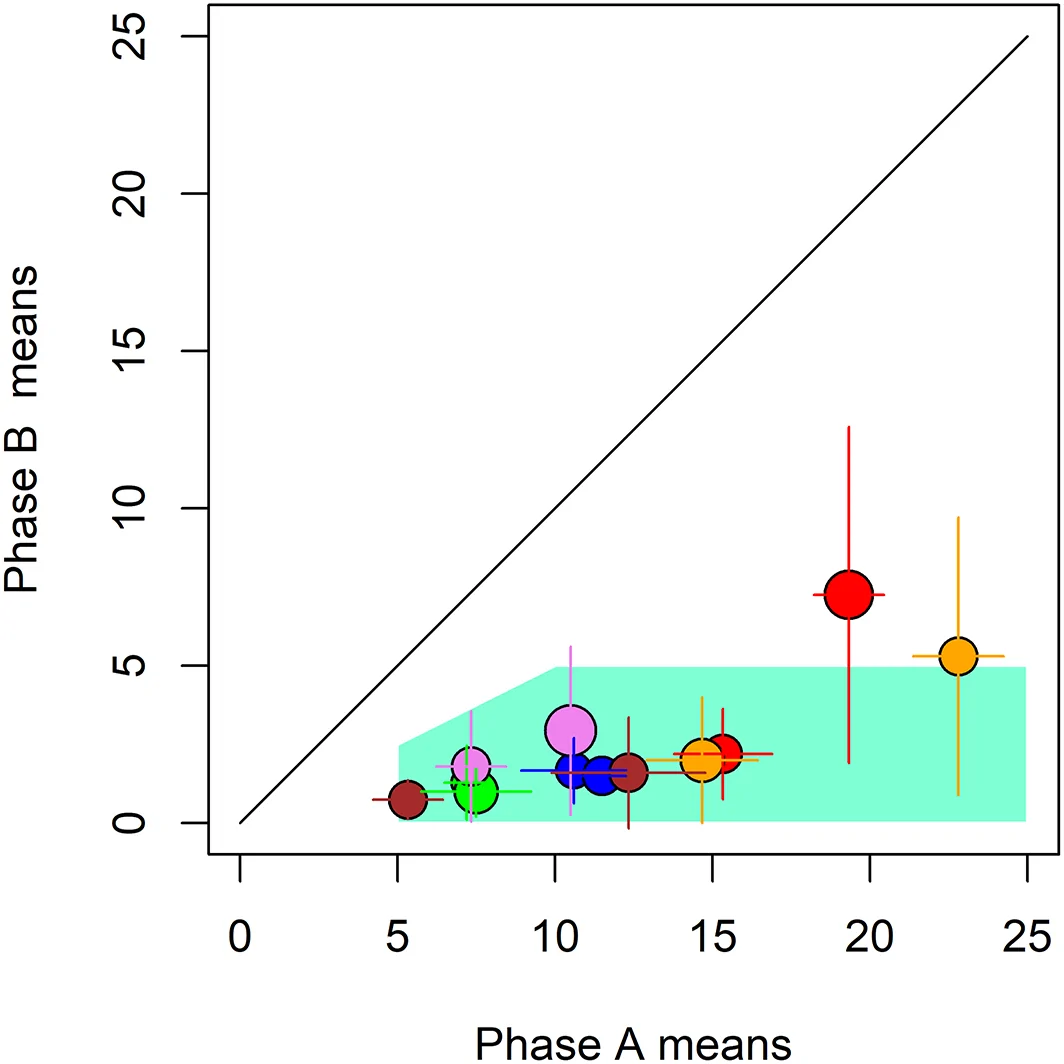
Modified Brinley Plot for Feeney and Ylvisaker (2003, 2006, 2008) *Note*. Each dot represents an A‐B comparison, with the x‐axis coordinate defined by the baseline mean and the y‐axis coordinate defined by the intervention phase mean. Dots of the same color belong to the same participant. The colored polygon represents effects with sufficient improvement and with a desired postintervention level of the target behavior. The size of the dot is proportional to the number of measurements in the A and B phases that are compared in the specific effect. The horizontal lines represent the lack of fit of the within‐phase mean to the baseline data. The vertical lines represent the lack of fit of the within‐phase mean to the intervention phase data.

Referring to both issues, Figure 7 illustrates how the number of measurements used to compute a mean difference and the degree of lack of fit of the means to the data can be represented on the modified Brinley plot. The larger the dots and the shorter the vertical and horizontal lines, the more reliable that these dots can be considered, understanding reliable as being based on more measurements (size of the dot) and representing the raw measurements (shortness of the lines around the dots).

#### The Mean is Not the Only Possible Summary

**Figure 8:**
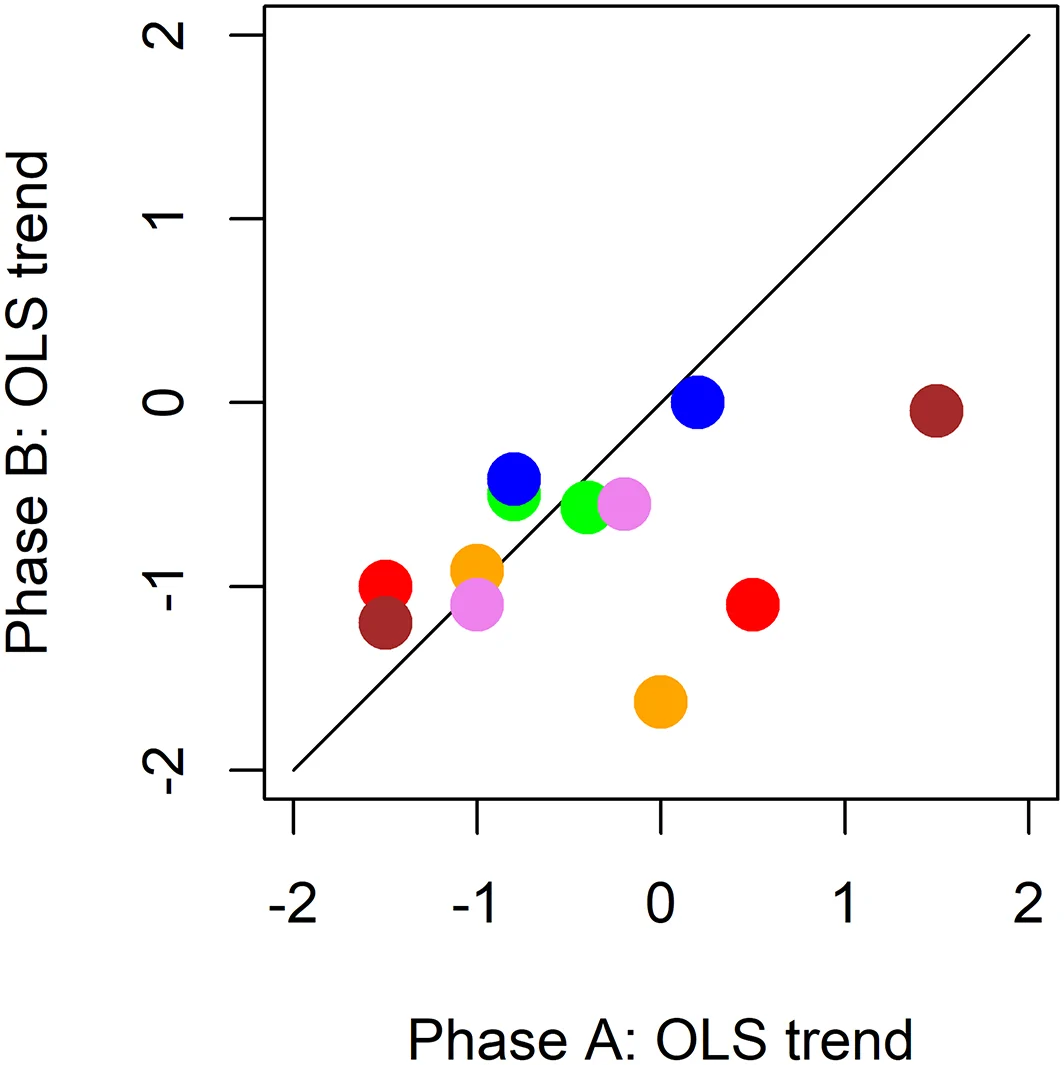
Modified Brinley Plot for Feeney and Ylvisaker (2003, 2006, 2008) *Note*. Each dot represents an A‐B comparison, with the x‐axis coordinate defined by the ordinary least squares estimate of the baseline trend and the y‐axis coordinate defined by ordinary least squares estimate of the intervention phase trend. Dots of the same color belong to the same participant.

A comparison in level is not the only way to assess the presence of an effect, given that other data features such as trend, variability, immediacy, and overlap are also relevant (Kratochwill et al., 2013; Lane & Gast, 2014; Ledford et al., 2019). Accordingly, in the modified Brinley plot, it is possible to represent other summary measures beyond the mean, for instance, an estimate of slope, the standard deviation, or the immediate effect (Manolov & Tanious, 2022). For instance, Figure 8 represents the ordinary least squares estimates of trend on the modified Brinley plot. Most of the dots are near the diagonal line, indicating similar trends in adjacent phases, except for three of the comparisons for which the Phase B trend is more negative (improving to a greater degree) than the previous Phase A trend.

When there is excessive variability or trends, the mean should not be used as the main summary measure when quantifying effects. Moreover, the mean is sensitive to outliers and a more resistant central tendency measure such as the median could theoretically be used. The illustrations in the current text are based on means because this is the original summary used in the modified Brinley plot (Blampied, 2017) and also because level is typically the object of SCED data analysis (Tanious & Onghena, 2021). However, this does not necessarily suggest that the mean is the optimal summary of central tendency (e.g., in the context of visual analysis, medians are commonly recommended; Lane & Gast, 2014).

Deciding what to quantify (e.g., change in level, change in slope, change in variability; immediate or delayed effect) must be related to the type of effect expected. This recommendation is commonly made in the context of randomization tests (Heyvaert & Onghena, 2014; Levin et al., 2017, 2021; Michiels et al., 2017), but also in general in terms of SCED data analysis (Manolov, Moeyaert, & Fingerhut, 2022).

#### The Modified Brinley Plot Should Not Be a Stand‐Alone Graph

Despite the previously mentioned possibilities for the modified Brinley plot (i.e., to represent the lack of fit of the mean line, to represent trend, to reflect which comparisons are based on more measurements), the current authors advocate for the use of this graphical representation alongside the typical time‐series graphs. There are several reasons for such a recommendation, when assessing the degree of replication of effects within a study or across multiple studies. First, the time‐series line graph must be included, at minimum for the sake of transparency (Aydin & Yassikaya, 2022; Tate et al., 2013). Second, although the modified Brinley plot can efficiently represent a summary for several individuals (across several studies), a time‐series plot can be useful for assessing how well these summaries (e.g., means, slopes of trend lines) fit the data. Third, the time‐series plot can inform about how change unfolds over time, whereas the modified Brinley plot allows for a more static image, which could be more useful for assessing consistency across and within participants (Manolov & Tanious, 2022).

If the results of multiple studies are to be integrated quantitatively as in a meta‐analysis, an additional graphical representation that can be used are the commonly employed forest plots (Fernández‐Castilla et al., 2020). The modified Brinley plot, as described here (with the original data, not standardized data), can be used when all target behaviors are measured in the same units, to represent both the preintervention and the postintervention level for each A‐B comparison. Complementing this graphical information, the forest plot can be used to represent a standardized measure of the size of the intervention effect in each study, alongside the confidence interval built around this effect size and the weight of the study in the overall summary measure.

## The Proposal

### Main Features: The Two Necessary Elements

In the current text we propose to use two previously mentioned graphical aids, represented on the modified Brinley plot, as building blocks for assessing the degree to which effects replicate. Specifically, the desired postintervention level (green dotted horizontal line on Figures 2, 4, and 6) and the desired amount of change after introducing the intervention (grey dashed diagonal line) define an area of the modified Brinley plot in which the dots should be placed if all meet both requirements (represented by a polygon).

The basis of the current proposal is that the two desired aspects (improvement and postintervention level) should be definable according to applied rather than statistical criteria (Kazdin, 2020). In the SCED context, the desired postintervention level^3^ can be specified a priori, as suggested in the context of the percentage of goal obtained (Ferron et al., 2020). Once criteria are available for what is desired, this would enable agreement in the interpretation of the results of the studies by authors. A dot within the polygon would be a desired effect and a dot outside of the polygon would be an insufficient or undesired effect. In general, the polygon can be used as a descriptive criterion regarding whether the basic effects are sufficiently replicated.

### Defining the Polygon Via Applied Criteria and Expert Judgment

The operative definition of “approximate replication,” “almost the same effect,” or “negligible difference” in effects is a matter of scientific judgment that is likely to be domain‐specific (Hedges & Schauer, 2019b). The definition of the polygon is related to applied significance, which can be related to aspects such as social comparisons (i.e., falling within a normative range), no longer meeting diagnostic criteria, or departure from dysfunctional behavior (Kazdin, 2020). Specifically, the desired postintervention level can be understood as falling within a normative range, whereas the desired amount of change can be understood as a sufficient departure from dysfunctional behavior, considering that this latter aspect has sometimes been assessed using quantitative/statistical criteria such as the reliable change index (Jacobson & Truax, 1991) and standard deviations (Kazdin, 2020). The need for both criteria can be related to the requirement of ending at an adequate level of the target behavior and having evidence that such level was not achieved before the intervention, but that there was a sufficient change associated with the introduction of the intervention.

The need for expert judgment is not a limitation, given that expert judgment is necessary when using a benchmark outcome or mastery criteria (Branch, 2014; Hagopian, 2020; Imam, 2021; Kazdin, 1977; McDougale et al., 2020; Perone, 1999; Shepley et al., 2020). Likewise, structured criteria for visual analysis do not substitute clinical judgment (Roane et al., 2013) and judgment is necessary when effect sizes are used to complement or substitute *p*‐values (Cortina & Landis, 2011). Thus, it has been recommended to interpret or label effect sizes in relation to the context of the study using expert judgment, both in the SCED context (Manolov et al., 2016; Vannest & Sallese, 2021) and outside of it (e.g., Dunst & Hamby, 2012; Durlak, 2009). Similarly, there is evidence that applied researchers are not fond of inspecting visually graphed data in the absence of context (Ford et al., 2020).

Specifically in relation to replication, even quantitative benchmarks for what is considered “almost the same” for effect are still social conventions among scientists (Hedges, 2019). In contrast to using domain‐specific knowledge, mindless application of statistical rituals is to be avoided (Gigerenzer, 2004). Thus, the exact way in which the Brinley polygon is defined is a matter of such domain‐specific knowledge, rather than an (impossibly) universally valid rule of thumb. This is not necessarily a limitation, but rather a distinctive feature of a scientific way of proceeding.

### Numerical Examples

**Figure 9:**
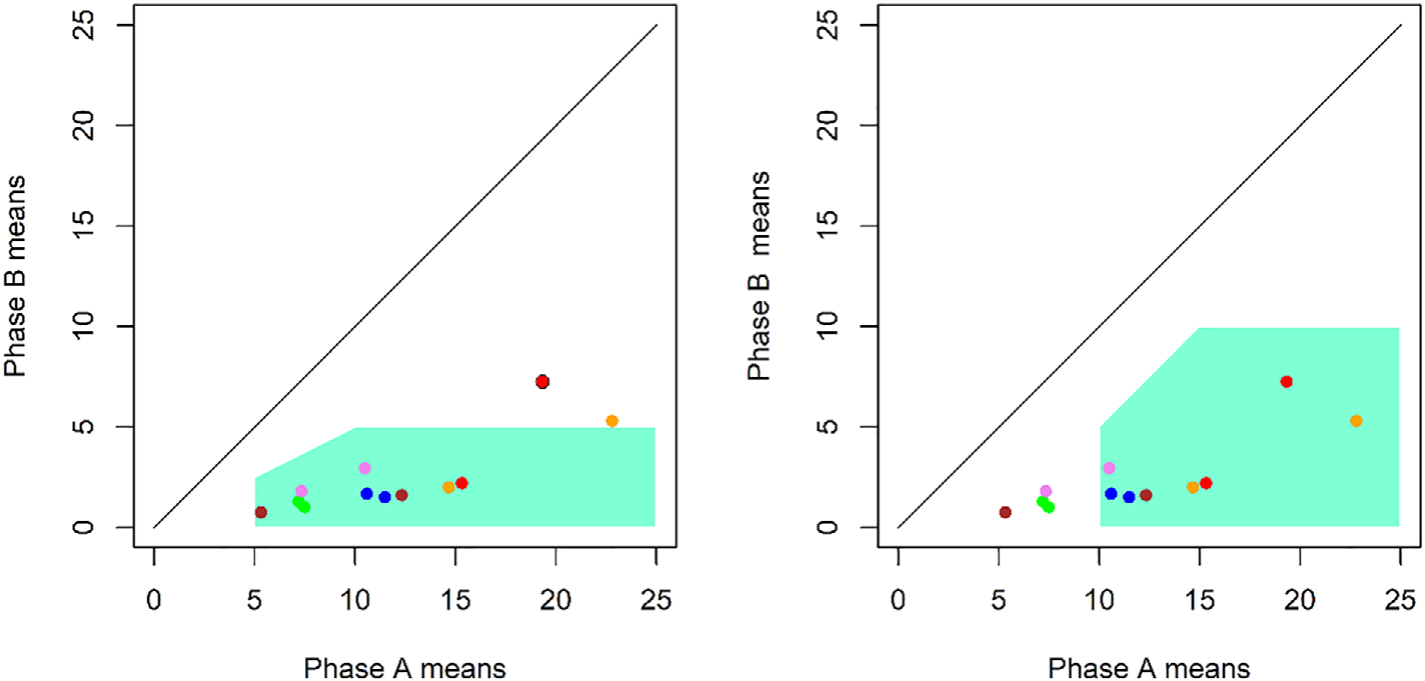
Modified Brinley Plots for Feeney and Ylvisaker (2003, 2006, 2008) *Note*. Each dot represents an A‐B comparison, with the x‐axis coordinate defined by the baseline mean and the y‐axis coordinate defined by the intervention phase mean. Dots of the same color belong to the same participant. The colored polygons represent effects with sufficient improvement and with a desired postintervention level of the target behavior.

In the current section, we will focus on the Feeney and Ylvisaker (2003, 2006, 2008) data for showing how the polygon is defined by specifying different values for the two necessary elements. The first example shown in Figure 6 included a desired postintervention level of a maximum of five aggressive behaviors and a minimum reduction of 50% with respect to the Phase A level. The corresponding polygon is represented on the left graph of Figure 9. Its upper side is defined by the maximal postintervention level of 5. Its lower side is defined by the minimal possible postintervention level of 0. Its right side is defined by the supposed maximal Phase A level^4^ of 25. Its left side is defined by a vertical line that would mark a Phase A level of 5 (on Figure 6 this is the red vertical line). If such a desired level is to be attributed to the intervention it should not take place already during Phase A. Therefore, the polygon is defined to the right of this vertical line. Finally, the diagonal line that cuts the rectangle and makes it a polygon of irregular shape is the grey dashed line from Figure 6 that marks the desired amount of change. For instance, for a Phase A level of 5, the desired 50% reduction would correspond to an intervention level of 2.5, whereas for a Phase A level of 10, the desired 50% reduction would correspond to an intervention level of 5. These are the two coordinates that define the diagonal side of the polygon. In terms of assessing replication with this polygon, 10 out of the 12 dots are within the polygon defined by the desired reduction and postintervention level, so the degree of replication is 10/12 = 83% (considering all effects depicted, within and across participants).

**Figure 10:**
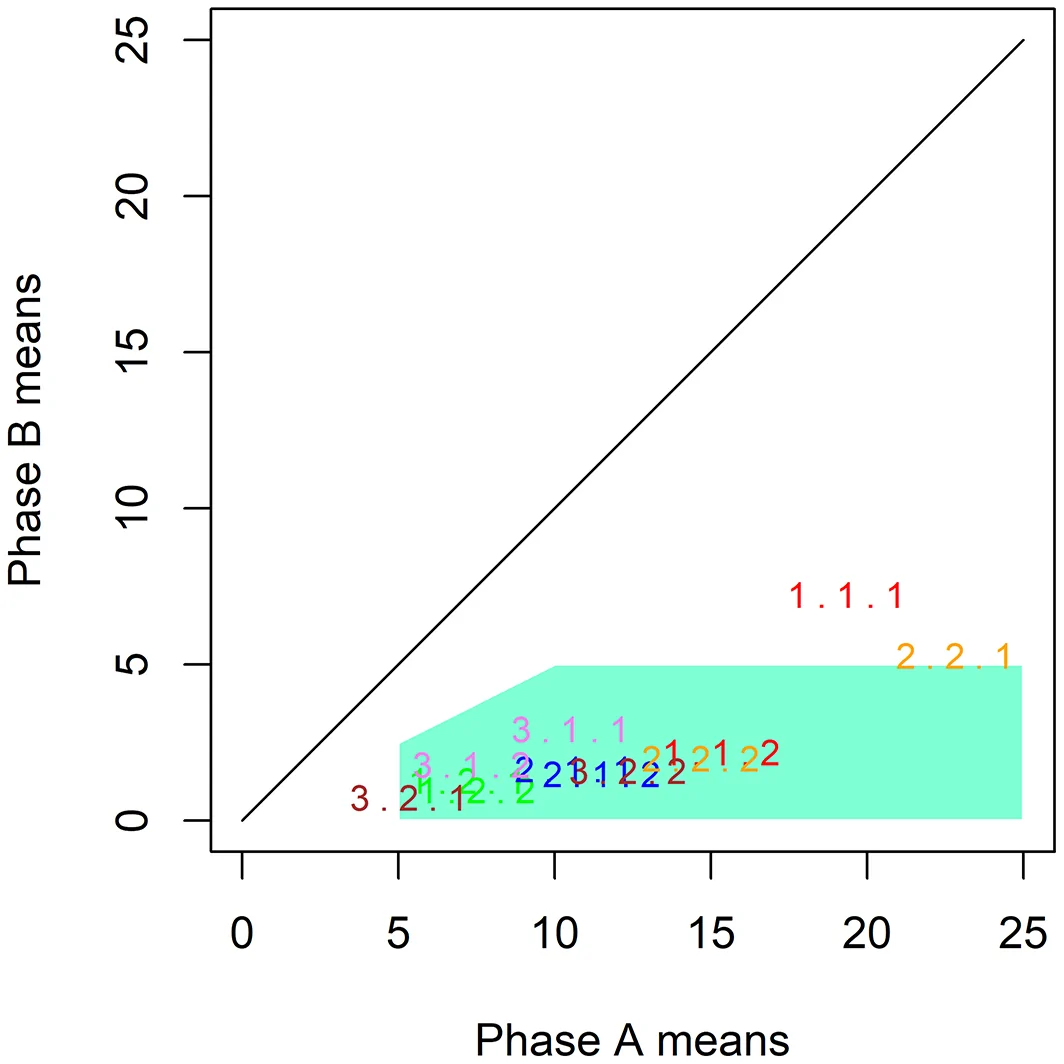
Modified Brinley Plot for Feeney and Ylvisaker (2003, 2006, 2008) *Note*. Each sequence of three digits represents an A‐B comparison, with the x‐axis coordinate defined by the baseline mean and the y‐axis coordinate defined by the intervention phase mean. The first digit represents the study, the second digit represents the participant within the study, and the third digit represents the A‐B comparison within the participant. The colored polygons represent effects with sufficient improvement and with a desired postintervention level of the target behavior.

Using the modified Brinley plot from Figure 10, it can be verified that the effects not included in the polygon are denoted by 1.1.1 and 2.2.1, which means that they refer to Study 1, Participant 1, first A‐B comparison, and Study 2, Participant 2, first A‐B comparison. The researcher can identify who these individuals are according to how the data file is organized, and also in relation to Figure 5. In that sense, the result of the assessment of replication is the proportion of effects within the desirable polygon. In summary, for each effect, a dichotomous decision is made regarding whether or not it is in the polygon. However, for all effects considered together, the decision is not dichotomous (effects replicated or not), but it is rather a quantification of the percentage of effects that are replicated, in the sense that they fall within the desired limits.

To better understand the construction of the polygon, another example is provided. Suppose that the maximal desired postintervention level is 10 and that the desired amount of change is five points (in absolute terms, not as a percentage). The corresponding polygon is represented in the right graph of Figure 9. Its upper side is defined by the maximal postintervention level of 10. As with the previous example, its lower side is defined by the minimal possible postintervention level, 0, and its right side is defined by the supposed maximum Phase A level of 25. The diagonal line that cuts the rectangle and makes it a polygon of irregular shape is the desired amount of change. For instance, for a Phase A level of 10, the desired reduction of five points would correspond to an intervention level of 5, whereas for a Phase A level of 15, the desired reduction of five points would correspond to an intervention level of 10. These are the two coordinates that define the diagonal side of the polygon in Figure 9b. In terms of assessing replication with this polygon, eight of the 12 dots are within it, suggesting a replication of 66.67%.

### Graphical Advantage of the Polygon

It was previously mentioned that the modified Brinley plot is not affected by the *x*:*y* ratio (Kubina et al., 2017), as it is square by definition. Moreover, the number of data points per *x*:*y* ratio (Radley et al., 2018) is also not critical when a visual aid such as the polygon is used for assessing replication, because this polygon (when printed sufficiently large) allows a visual inspection of the dots that are and are not included. Thus, these elements of graphical display, which can be potentially distorting for time‐series graphs, are not expected to affect the replication polygon.

### Methodological Framework: In‐depth Description and Rationale for the Proposal

#### Similar Assessment of Replication Within and Across Studies

The polygon represented on the modified Brinley plot allows for the assessment of replication at three levels: (a) within a participant for an ABAB design (and extensions of it), an alternating treatments design, or a multiple‐baseline design across behaviors or settings; (b) across participants in a multiple‐baseline design across subjects; and (c) across studies. This is well‐aligned with Schauer et al. (2021)), who state that “Patterns used to describe replication across multiple findings should be somewhat consistent with the definitions used to define replication for a single finding” (p. 18).

#### Preserving the Individual Level of Analysis

The proposal for assessing replication is well‐aligned with Hagopian's (2020) recommendation to examine findings within and across participants in a manner that preserves the analysis of individual outcomes. Specifically, the modified Brinley plot with the polygon plots each individual case as a distinct data point, representing outcomes across the collective and documenting their distribution, as suggested. Similarly, the result of the evaluation of replication is in accordance with another recommendation made by Hagopian, namely, that outcomes across participants are described in terms of the percentage of cases where certain outcomes were obtained rather than averages.

#### Approximate Replication and Conceptualization of Variability

From a methodological perspective, the proposal can be used for both intra‐ and intersubject replications, taking place within or across studies. The modified Brinley plot is applicable to direct replications as opposed to systematic replications, because varying certain variables (and not merely replicating with different participants) is likely to entail greater variability in the effects and the need to consider moderating effects, which is not possible in the context of the modified Brinley plot. In that sense, from a meta‐analytical across‐studies perspective, the modified Brinley plot can be applied to represent a fixed‐effect model (assuming all studies estimate the same population effect) rather than a random‐effects model (assuming different studies estimate a different population effect).

From a quantitative perspective, the focus is placed on approximate replication (or practical equivalence) instead of exact replication (i.e., effects that are equal in magnitude). Thus, the variability of the dots within the polygon would be conceptualized as negligible differences (Hedges & Schauer, 2019a) or negligible heterogeneity (Hedges & Schauer, 2019b). Within this polygon, there is no qualitative disagreement between the effects (Hedges & Schauer, 2019a). Similarly, it must be highlighted that variability is a natural and expected phenomenon that does not preclude generalization (De Luca Picione, 2015).

It should be noted that the variability is not summarized as a single value. Options for single‐value summaries include the Mean Euclidean Distance between all pairs of data points (like the study of diversity of groups, conceptualized as separation; Biemann & Kearney, 2010; Harrison & Klein, 2007), as well the *p*‐value resulting from the Q‐test commonly used in meta‐analysis (Hedges & Schauer, 2019a, 2019b). However, it is not only important how similar the effects are, but around which values they are similar, as the polygon focuses on the specific values that are considered of applied or practical relevance. Similarly, interindividual differences are not treated solely as departures from the overall mean. Instead, all effects for all individuals are depicted in the modified Brinley plot and thus individual summary data (but not raw measurements) are readily available in the modified Brinley plot.

#### There is No Specific Target Study and No Putative Population of Studies

The current proposal does not follow the framework of target study versus replication due to its limitations (Hedges & Schauer, 2019b). In contrast, it allows for the assessment of the variability of effects in a body of evidence, instead of validating one result. Therefore, the proposal can be understood as a groupwise rather than a pairwise method (Schauer et al., 2021). However, focusing on the data available is consistent with a fixed‐effect framework (Hedges & Schauer, 2019a; Hedges & Vevea, 1998). What is studied is the similarity in (or agreement between) the observed studies, which are considered the whole population of interest.

### Limitations and Challenges when Using the Proposal

#### What the Proposal is Not About

The current proposal is not intended to be used, and cannot be used, to determine how many replications are necessary (Hitchcock et al., 2015; Kratochwill et al., 2013; Lanovaz & Rapp, 2016; Lanovaz & Turgeon, 2020) or whether any replications at all are necessary in a given context (Lanovaz et al., 2019). Moreover, we do not make any specific quantitative proposals for how much improvement should be considered necessary, when defining the polygon or whether this improvement should be expressed in absolute terms (e.g., number of behaviors, points in an inventory) or in relative terms (e.g., 25% increase or 50% reduction in comparison to the Phase A level). As stated previously, this is a matter of domain‐specific expert judgment, and it cannot be superseded by an arbitrary statistical criterion imposed from outside of the domain in absence of a wide consensus.

Due to the way in which the polygon is defined, the proposal is mainly focused on the magnitude of effect when comparing a baseline (Phase A) to an intervention Phase (B). In that sense, the modified Brinley plot does not include a representation of (or information about) maintenance or generalization. In general, an effect that is replicated, according to the Brinley polygon, is not necessarily an effect that is socially valid. Thus, just as visual inspection of a time‐series line graph cannot substitute for the assessment for social validity, we do not propose the Brinley polygon as a substitute for assessing the multiple different aspects related to social validity (see Horner et al., 2005). We only propose the polygon for assessing replication. However, it must be highlighted that the Brinley polygon is related to two of the aspects relevant for social validity. The desired postintervention level is potentially related to normative comparisons (Snodgrass et al., 2018). However, the desired amount of change from the Phase A level is related to having an effect of sufficient magnitude for achieving social importance (Spear et al., 2013).

#### Applied Criteria May Be Difficult to Derive

Regarding establishing the desired postintervention level, a definition of a normative range is not always easy and diagnostic criteria may not always be susceptible to being translated to simple cut‐off points (Kazdin, 2020). Moreover, the polygon (or any other statistical or visual tool, for that matter) may not necessarily reflect the subjective evaluation by the participant regarding whether the intervention introduced a change in their life (Kazdin, 2020).

Regarding establishing the desired amount of change from the Phase A, a priori, it is equivalent to specifying the minimum difference criterion in the technically more complex and inferential probability of replication (Sanabria & Killeen, 2007). This step may not always be straightforward. In certain situations, it would make sense to first familiarize oneself with the Phase A level (i.e., gather data), before determining how much of a change would be sufficient. Deciding the desired intervention level according to the observed Phase A level and variability is recommended for changing criterion designs (Hartmann & Hall, 1976). However, in the context of the polygon, the desired amount of change cannot be determined individually for each A‐B comparison according to the Phase A data, because this would entail multiple polygons. The alternative is to rely on previous research, for identifying the amount of change (e.g., as a percentage) that has been deemed sufficient. This would be a better alternative than establishing arbitrary amounts such as 25%, 33%, or 50%, without any justification. In the event the researcher is unable to define any desired amount of change, it is possible to take an extreme stance and consider any nonzero difference (e.g., 1%) in the expected direction as sufficient. This would be analogous to the initial definition of replication in the framework of the probability of replication: “Define replication as an effect of the same sign^5^ as that found in the original experiment” (Killeen, 2005, p. 346). It would also approximate a reductionist interpretation of the questions included for systematic protocols for the visual analysis of time‐series graphs, for instance, “Is there an overall level change between baseline and treatment phases?” (Maggin et al., 2013, p. 56) and “Is there an immediate change from the last 3‐5 data points in baseline to the first 3‐5 data points in treatment?” (Wolfe et al., 2019, p. 495). In these questions, the undefined word “change” can be interpreted as any kind of change. Such a definition of the desired amount of change from the Phase A would make the polygon mostly dependent on the desired postintervention level.

#### Avoiding Questionable Research Practices

Ideally, the polygon must be defined prior to gathering or inspecting the data (mean levels in case of the modified Brinley plot), in accordance with the principles of preregistration and transparency (Ariens et al., 2020; Johnson & Cook, 2019; Porcino et al., 2020) and the importance of a priori decisions in data analysis (Manolov, Moeyaert, & Fingerhut, 2022). However, an exception could take place in situations in which it might be required to know the Phase A level first. In any case, the definition must be accompanied with a justification, just as justifications are required when choosing an option for the main quantitative analysis of the data (Tate et al., 2013).

The aim of such a priori documented decisions is to avoid questionable research practices (Laraway et al., 2019), such as defining the polygon a posteriori, once all the data are available and depicted on the modified Brinley plot. The requirement for an a priori specification of the polygon is similar to defining mastery criteria (Shepley et al., 2020) or the rules for shifting phases (Kazdin, 2020) before data collection. The main issues that can arise in terms of questionable research practices are: (a) if there is no preregistration of the two elements determining the polygon, or (b) if the researchers consider that it is impossible to define these two elements beforehand. Either of these issues would be a problem only if researchers purposely manipulated the polygon after the data were gathered to achieve a replicated effect. However, such a problem taking place would require assuming intentional bad research practice, combined with capacity to justify a posteriori the definition of the desired postintervention level and the desired amount of change from the Phase A level. In contrast, in the presence of certain criteria for assessing the social validity of the intervention effect (e.g., sufficient magnitude of change, normative range), these same criteria can be used for defining the polygon a priori and avoiding questionable research practices.

### Software Use

#### Preparing the Data File

**Figure 11:**
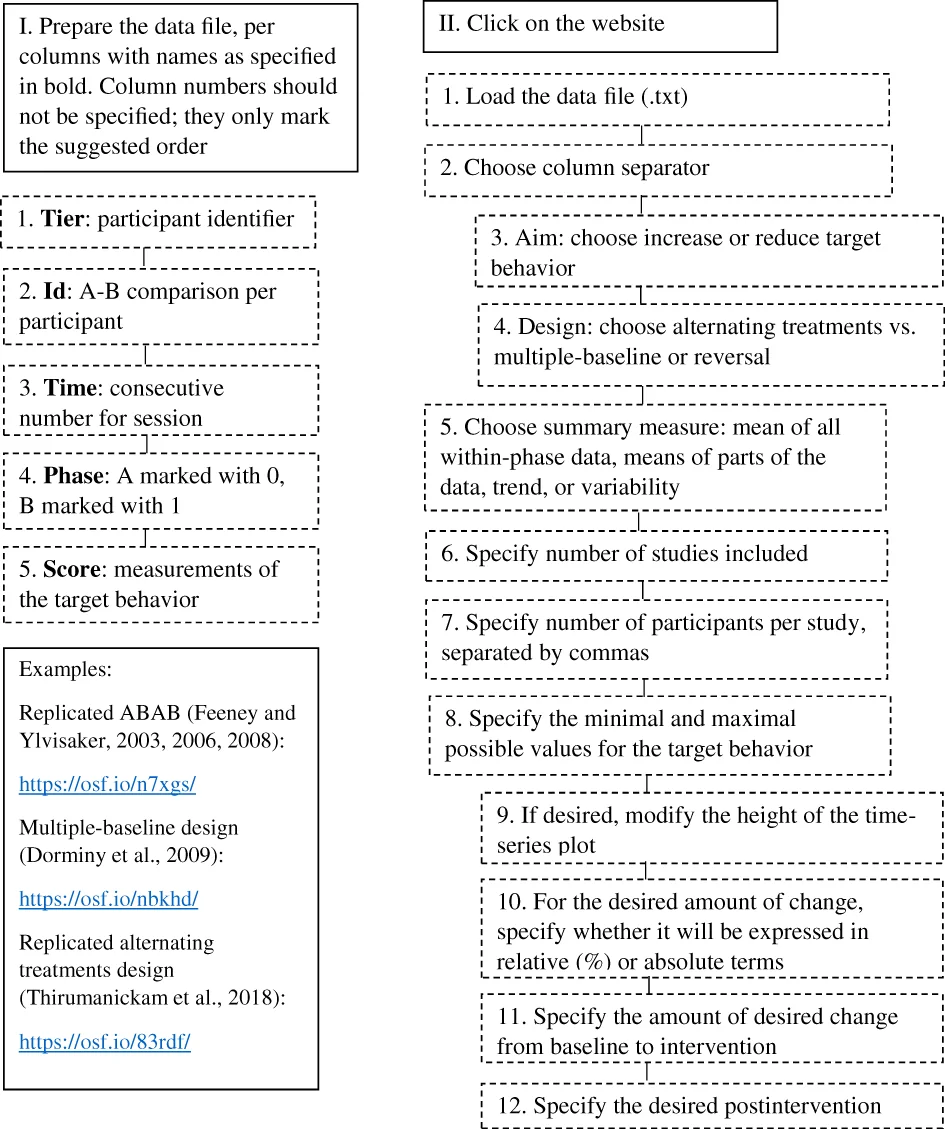
Task Analysis for Using the Developed Software

A free user‐friendly website can be used for obtaining the polygon (https://manolov.shinyapps.io/Brinley/) and it was used for obtaining all modified Brinley plots presented in the current text. To use the software, it is necessary to prepare a data file (e.g., via a program such as Microsoft Excel) with the structure illustrated on the website itself. Figure 11 contains the characteristics of the data file and the steps required for using the website.

We contend that the amount of effort required of applied researchers for obtaining the replication polygon is rather low once the desired postintervention level and the desired amount of change from the Phase A level are determined. To obtain any graphical representation of SCED data, it is necessary to organize the data in a data file, thus, this is not an added difficulty. Afterwards, to use the website, it is not necessary to download, install, or learn to use any new software. The researcher makes choices by clicking or by writing a few numbers but nothing else is required. The software is an application built via Shiny, as for other data analytical approaches (e.g., Declercq et al., 2020; Kranak et al., 2021), which makes training of users to construct the graphs for themselves following multiple steps unnecessary (e.g., Dixon et al., 2009; Lehardy et al., 2021; Mitteer et al., 2018).

## Discussion

The purpose of the current paper is to present and justify a visual approach towards assessing whether or not the results of different replications of A‐B comparisons agree. To the best of our knowledge, this is the first proposal dealing specifically with the assessment of replication within and across SCED studies, in terms of how it can be objectively performed, while giving priority to expert judgment over arbitrary statistical criteria. This visual assessment is guided by a priori criteria on the desired effect of the intervention, and it is aided by introducing graphical elements to the modified Brinley plot (Blampied, 2017). The assessment of the degree of replication can be performed both within the context of a single study (e.g., when there are several participants, as in a multiple‐baseline design) or across studies (e.g., in similar situations to the ones in which a fixed‐effect meta‐analysis is performed). In contrast to other possible approaches to studying replication (e.g., *p*_rep_ by Killeen, 2005, or Q‐test, Hedges & Schauer, 2019b), the proposal is simple, visual, and focused on the magnitude of effect, which is especially relevant when working with few participants, making inferential statistics not applicable.

### Implications of the Use of the Polygon

In relation to the way in which the polygon is defined, it is compulsory that applied researchers establish (and report) criteria for a successful intervention prior to gathering the data. This is well‐aligned with current recommendations for avoiding confirmation bias (Laraway et al., 2019; Levin et al., 2017; Manolov, Moeyaert, & Fingerhut, 2022). The modified Brinley plot and the superimposed polygon allow for a wider perspective on the effect of the intervention, across A‐B comparisons and across participants. This perspective retains part of the information from the raw measurements and does not entail a reduction to a mere average or standard deviation (Normand, 2016).

The emphasis for establishing intervention generality should not be solely on counting the number of successful replications of an intervention effect or on requiring a 3:1 ratio of effects to noneffects (Cook et al., 2015). Instead, the focus can be placed on the similarities and differences in successful and unsuccessful replications (Maggin, 2015). Researchers can be encouraged to include homogeneous studies in terms of participants, interventions, and study characteristics, for which replication of the effect can be logically expected. However, once an effect that is not within the polygon representing the desired effect is present, it is possible to investigate the reasons for this failure to replicate results to understand for whom and why the results did not replicate or generalize (Kazdin, 2020). Researchers should review that study to see whether there is any study, participant, or intervention characteristic that differs from the other studies. Thus, looking at moderator variables^6^ as the sources of variability or lack of replication is important to understand better the behavioral processes (Barton et al., 2016) and what works for whom (Ledford et al., 2016; Tincani & Travers, 2018). Such an endeavor is possible, even when working with summaries such as means instead of raw measurements.

Finally, it should be noted that although the assessment of the degree of replication is apparently performed in a purely visual way, there are several underlying quantifications. The desired change and the desired goal are quantities that are represented via the polygon. However, the dots and their additional graphical elements (i.e., size and error bars) are based on quantifications. This is well‐aligned with: (a) the emphasis on the complementarity between visual and quantitative analysis (Harrington & Velicer, 2015; Karazsia, 2018; Maggin et al., 2019), (b) the inherent use of quantifications when performing visual analysis (Lane & Gast, 2014), and (c) the fact that even visual aids such as the conservative dual criterion (Fisher et al., 2003) and the two‐standard deviations band (Pfadt & Wheeler, 1995) are based on statistical probability models.

### Additional Graphical Elements: Less is More?

Overall, it could be argued that additional graphical elements such as the size of the dot and the presence of error bars add noise or complexity to the modified Brinley plot. If there are many effects represented and an unclear intertwining of errors bars, this could certainly be the case. Nonetheless, there are two aspects to consider: (a) the software implementation of the proposal (https://manolov.shinyapps.io/Brinley/) includes both graphical representations without these additional elements (as Figure 9) and with them (as Figure 7), and (b) these elements are informative and can indicate whether more caution is needed when interpreting some of the effects. Specifically, in the event the effects (i.e., dots) are included in the replication polygon, but their error bars cross the borders of this polygon, this would suggest that the variability surrounding the summary measures (means, converted to dots) sheds some doubt on the researchers' confidence that the effects are clearly replicated. Thus, such additional elements can help provide more nuanced interpretations.

In terms of the informative value of the additional graphical elements, in the example of Figure 7, it is noteworthy that the two dots that are outside of the replication polygon are the ones associated with more error (or less precision) in terms of how the Phase B mean represents the data from this phase. In contrast, the dots that are included in the replication polygon show much smaller error vertical and horizontal error bars. In that sense, the fact that most of the dots are (descriptively) within the polygon can be trusted to a greater degree than the fact that there are two dots outside of the polygon. Moreover, the rightmost dot that is just above the upper border of the polygon is based on relatively fewer measurements and this adds further caution to the conclusion that this effect is not replicated.

In terms of the informative value of the additional graphical elements in general, one of the advantages of visual analysis is the possibility to take into consideration several data aspects at the same time (Parker et al., 2006), as is commonly recommended (Kratochwill et al., 2013; Ledford, Barton, Severini, & Zimmerman, 2019; Maggin et al., 2018). Even visual aids entail more than one data aspect. For instance, the conservative dual criterion (Fisher et al., 2003) involves representing mean and trend, and the application of statistical process control (Callahan & Barisa, 2005; Pfadt & Wheeler, 1995) involves representing mean and variability. Similarly, a proposal for making the functional analysis of behavior automatic also entails multiple data features (Kranak et al., 2021). Additionally, in a forest plot as a graphical representation commonly used in meta‐analyses, the size of the dot or square representing an effect reflects its precision and is related to the number of measurements that it is based on (Anzures‐Cabrera & Higgins, 2010). In that sense, the current proposal to represent variability around the mean and the number of measurements that an effect is based on is well aligned with current practices. Extending common visual representations has also been topic of recent research (e.g., Fernández‐Castilla et al., 2020; Snodgrass et al., 2022).

### Future Research

The main limitations of the proposal were already outlined previously. Regarding the current text, we focused on presenting the extension of the modified Brinley plot and provide a rationale for it, in relation to the recent developments and the importance of replication. We also provided an illustration with real behavioral data, but we did not perform a field test or a comparison with alternative ways of assessing replication. In that sense, a potential line for future research is to include a set of studies on the same target behavior with the same intervention (e.g., as identified in a research synthesis or a meta‐analysis) and to compare the conclusions drawn thanks to the polygon with other possible criteria for evaluating whether each A‐B comparison can be considered to represent an effect and regarding the degree to which these effects replicate (i.e., fall in the same category). Some of these possible criteria for assessing each A‐B comparison could be: (a) empirically based interpretative benchmarks for quantifications of effect (e.g., Harrington & Velicer, 2015; Parker & Vannest, 2009); (b) subjective evaluation of the participants or their significant ones, as part of the assessment of social validity (Snodgrass et al., 2018); or (c) whether the confidence interval for each effect includes zero or not. Another possible line of future research is to test the proposal with applied researchers. Such a test would inform about the degree to which researchers consider that the tool provides useful information and the degree to which it is easy to use.

## Footnotes

^1^
In De Luca Picione's (2015) words, “Psychology has considered nomothetic knowledge as the search for laws valid for groups and aggregations, rather than the search for laws applicable to all people” (p. 363).

^2^
For data analytical options applicable to different kinds of alternating treatment designs the interested reader can consult Manolov and Onghena (2018) and Manolov, Tanious, and Onghena (2022).

^3^
When visually inspecting the raw data in a time series plot, it can also be useful to depict the desired amount of progress until the final level is reached (Wolfe et al., 2021), equivalent to a goal line (Riley‐Tillman et al., 2020). However, this option is not used for the current proposal, as the modified Brinley plot works with summaries rather than with raw data.

^4^
Alternatively, the largest value (or mean) observed in either phase could be used.

^5^
Meaning in the same direction (i.e., that one condition is always superior to the other).

^6^
For a review of commonly used moderator variables in meta‐analysis, consult Moeyaert et al. (2021)

## References

[jaba923-bib-0001] Anzures‐Cabrera, J. , & Higgins, J. P. (2010). Graphical displays for meta‐analysis: An overview with suggestions for practice. Research Synthesis Methods, 1(1), 66‐80. 10.1002/jrsm.6 26056093

[jaba923-bib-0002] Ariens, S. , Ceulemans, E. , & Adolf, J. K. (2020). Time series analysis of intensive longitudinal data in psychosomatic research: A methodological overview. Journal of Psychosomatic Research, 137, 110191. 10.1016/j.jpsychores.2020.110191 32739633

[jaba923-bib-0003] Aydin, O. , & Yassikaya, M. Y. (2022). Validity and reliability analysis of the PlotDigitizer software program for data extraction from single‐case graphs. Perspectives on Behavior Science, 45(1), 239‐257. 10.1007/s40614-021-00284-0 PMC8894524 35342869

[jaba923-bib-0004] Barton, E. E. , Ledford, J. R. , Lane, J. D. , Decker, J. , Germansky, S. E. , Hemmeter, M. L. , & Kaiser, A. (2016). The iterative use of single case research designs to advance the science of EI/ECSE. Topics in Early Childhood Special Education, 36(1), 4‐14. 10.1177/0271121416630011

[jaba923-bib-0005] Biemann, T. , & Kearney, E. (2010). Size does matter: How varying group sizes in a sample affect the most common measures of group diversity. Organizational Research Methods, 13(3), 582‐599. 10.1177/1094428109338875

[jaba923-bib-0006] Bishara, A. J. , Peller, J. , & Galuska, C. M. (2021). Misjudgment of interrupted time‐series graphs due to serial dependence: Replication of Matyas and Greenwood (1990). Judgment and Decision Making, 16(3), 687‐708. http://journal.sjdm.org/20/200728d/jdm200728d.pdf

[jaba923-bib-0007] Blampied, N. M. (2017). Analyzing therapeutic change using modified Brinley plots: History, construction, and interpretation. Behavior Therapy, 48(1), 115‐127. 10.1016/j.beth.2016.09.002 28077215

[jaba923-bib-0008] Branch, M. (2014). Malignant side effects of null‐hypothesis significance testing. Theory & Psychology, 24(2), 256‐277. 10.1177/0959354314525282

[jaba923-bib-0009] Brown, C. L. , Bosley, H. G. , Kenyon, A. D. , Chen, K. H. , & Levenson, R. W. (2019). An idiographic statistical approach to clinical hypothesis testing for routine psychotherapy: A case study. Behaviour Research and Therapy, 118(July), 43‐53. 10.1016/j.brat.2019.03.014 30991265

[jaba923-bib-0010] Byiers, B. J. , Pennington, B. , Rudolph, B. N. , & Ford, A. L. (2021). Perspectives on the use of quantitative analysis in single‐case experimental research. Journal of Behavioral Education, 30(3), 444‐454. 10.1007/s10864-020-09386-2

[jaba923-bib-0011] Callahan, C. D. , & Barisa, M. T. (2005). Statistical process control and rehabilitation outcome: The single‐subject design reconsidered. Rehabilitation Psychology, 50(1), 24‐33. 10.1037/0090-5550.50.1.24

[jaba923-bib-0012] Caneiro, J. P. , Smith, A. , Linton, S. J. , Moseley, L. , & O'Sullivan, P. (2019). ‘How does change unfold?’ An evaluation of the process of change in four people with chronic low back pain and high pain‐related fear managed with Cognitive Functional Therapy: A replicated single‐case experimental design study. Behaviour Research and Therapy, 117(June), 28‐39. 10.1016/j.brat.2019.02.007 30853096

[jaba923-bib-0013] Cook, B. G. , Buysse, V. , Klingner, J. , Landrum, T. J. , McWilliam, R. A. , Tankersley, M. , & Test, D. W. (2015). CEC's standards for classifying the evidence base of practices in special education. Remedial and Special Education, 36(4), 220‐234. 10.1177/0741932514557271

[jaba923-bib-0014] Cortina, J. M. , & Landis, R. S. (2011). The Earth is not round (p = .00). Organizational Research Methods, 14(2), 332‐349. 10.1177/1094428110391542

[jaba923-bib-0015] Cumming, G. (2008). Replication and p intervals: P values predict the future only vaguely, but confidence intervals do much better. Perspectives on Psychological Science, 3(4), 286‐300. 10.1111/j.1745-6924.2008.00079.x 26158948

[jaba923-bib-0016] Declercq, L. , Cools, W. , Beretvas, S. N. , Moeyaert, M. , Ferron, J. M. , & Van den Noortgate, W. (2020). MultiSCED: A tool for (meta‐)analyzing single‐case experimental data with multilevel modeling. Behavior Research Methods, 52(1), 177‐192. 10.3758/s13428-019-01216-2 30972557

[jaba923-bib-0017] De Luca Picione, R. (2015). The idiographic approach in psychological research. The challenge of overcoming old distinctions without risking to homogenize. Integrative Psychological and Behavioral Science, 49(3), 360‐370. 10.1007/s12124-015-9307-5 25939530

[jaba923-bib-0018] DeRosa, N. M. , Sullivan, W. E. , Roane, H. S. , & Kadey, H. J. (2021). Single‐case experimental designs. In W. W. Fisher , C. C. Piazza , & H. S. Roane (Eds.), Handbook of applied behavior analysis (2nd ed., pp. 155‐171). The Guilford Press.

[jaba923-bib-0019] Dixon, P. , & Glover, S. (2020). Assessing evidence for replication: A likelihood‐based approach. Behavior Research Methods, 52(6), 2452‐2459. 10.3758/s13428-020-01403-6 32441033

[jaba923-bib-0020] Dixon, M. R. , Jackson, J. W. , Small, S. L. , Horner‐King, M. J. , Lik, N. M. , Garcia, Y. , & Rosales, R. (2009). Creating single‐subject design graphs in Microsoft Excel 2007. Journal of Applied Behavior Analysis, 42(2), 277‐293. 10.1901/jaba.2009.42-277 PMC2695331 19949515

[jaba923-bib-0021] Dorminy, K. P. , Luscre, D. , & Gast, D. L. (2009). Teaching organizational skills to children with high functioning autism and Asperger's syndrome. Education and Training in Developmental Disabilities, 44(4), 538‐550. https://www.jstor.org/stable/24234261

[jaba923-bib-0022] Dowdy, A. , Peltier, C. , Tincani, M. , Schneider, W. J. , Hantula, D. A. , & Travers, J. C. (2021). Meta‐analyses and effect sizes in applied behavior analysis: A review and discussion. Journal of Applied Behavior Analysis, 54(4), 1317‐1340. 10.1002/jaba.862 34219222

[jaba923-bib-0023] Dunst, C. J. , & Hamby, D. W. (2012). Guide for calculating and interpreting effect sizes and confidence intervals in intellectual and developmental disability research studies. Journal of Intellectual & Developmental Disability, 37(2), 89‐99. 10.3109/13668250.2012.673575 22530580

[jaba923-bib-0024] Durlak, J. A. (2009). How to select, calculate, and interpret effect sizes. Journal of Pediatric Psychology, 34(9), 917‐928. 10.1093/jpepsy/jsp004 19223279

[jaba923-bib-0025] Etz, A. , & Vandekerckhove, J. (2016). A Bayesian perspective on the reproducibility project: Psychology. PLOS One, 11(2), e0149794. 10.1371/journal.pone.0149794 PMC4769355 26919473

[jaba923-bib-0026] Fahmie, T. A. , & Hanley, G. P. (2008). Progressing toward data intimacy: A review of within‐session data analysis. Journal of Applied Behavior Analysis, 41(3), 319‐331. 10.1901/jaba.2008.41-319 PMC2521866 18816972

[jaba923-bib-0027] Falligant, J. M. , McNulty, M. K. , Hausman, N. L. , & Rooker, G. W. (2020). Using dual‐criteria methods to supplement visual inspection: Replication and extension. Journal of Applied Behavior Analysis, 53(3), 1789‐1798. 10.1002/jaba.665 31851379

[jaba923-bib-0028] Feeney, T. J. , & Ylvisaker, M. (2003). Context‐sensitive behavioral supports for young children with TBI: Short‐term effects and long‐term outcome. The Journal of Head Trauma Rehabilitation, 18(1), 33‐51. 10.1097/00001199-200301000-00006. 12802236

[jaba923-bib-0029] Feeney, T. , & Ylvisaker, M. (2006). Context‐sensitive cognitive‐behavioural supports for young children with TBI: A replication study. Brain Injury, 20(6), 629‐645. 10.1080/02699050600744194 16754288

[jaba923-bib-0030] Feeney, T. J. , & Ylvisaker, M. (2008). Context‐sensitive cognitive‐behavioral supports for young children with TBI: A second replication study. Journal of Positive Behavior Interventions, 10(2), 115‐128. 10.1177/1098300707312540

[jaba923-bib-0031] Fernández‐Castilla, B. , Declercq, L. , Jamshidi, L. , Beretvas, N. , Onghena, P. , & Van den Noortgate, W. (2020). Visual representations of meta‐analyses of multiple outcomes: Extensions to forest plots, funnel plots, and caterpillar plots. Methodology, 16(4), 299‐315. 10.5964/meth.4013

[jaba923-bib-0032] Ferron, J. M. , Goldstein, H. , Olszewski, A. , & Rohrer, L. (2020). Indexing effects in single‐case experimental designs by estimating the percent of goal obtained. Evidence‐Based Communication Assessment and Intervention, 14(1‐2), 6‐27. 10.1080/17489539.2020.1732024

[jaba923-bib-0033] Ferron, J. M. , Joo, S.‐H. , & Levin, J. R. (2017). A Monte Carlo evaluation of masked visual analysis in response‐guided versus fixed‐criteria multiple‐baseline designs. Journal of Applied Behavior Analysis, 50(4), 701‐716. 10.1002/jaba.410 28887866

[jaba923-bib-0034] Fife, D. A. , Longo, G. , Correll, M. , & Tremoulet, P. D. (2021). A graph for every analysis: Mapping visuals onto common analyses using flexplot. Behavior Research Methods, 53(5), 1876‐1894. 10.3758/s13428-020-01520-2 33634423

[jaba923-bib-0035] Fisher, W. W. , Kelley, M. E. , & Lomas, J. E. (2003). Visual aids and structured criteria for improving visual inspection and interpretation of single‐case designs. Journal of Applied Behavior Analysis, 36(3), 387‐406. 10.1901/jaba.2003.36-387 PMC1284456 14596583

[jaba923-bib-0036] Ford, A. L. , Rudolph, B. N. , Pennington, B. , & Byiers, B. J. (2020). An exploration of the interrater agreement of visual analysis with and without context. Journal of Applied Behavior Analysis, 53(1), 572‐583. 10.1002/jaba.560 30924129

[jaba923-bib-0037] Gigerenzer, G. (2004). Mindless statistics. Journal of Socio‐Economics, 33(5), 587‐606. 10.1016/j.socec.2004.09.033

[jaba923-bib-0038] Hagopian, L. P. (2020). The consecutive controlled case series: Design, data‐analytics, and reporting methods supporting the study of generality. Journal of Applied Behavior Analysis, 53(2), 596‐619. 10.1002/jaba.691 PMC8805508 32125716

[jaba923-bib-0039] Hantula, D. A. (2019). Editorial: Replication and reliability in behavior science and behavior analysis: A call for a conversation. Perspectives on Behavior Science, 42(1), 1‐11. 10.1007/s40614-019-00194-2 PMC6701722 31976418

[jaba923-bib-0040] Harrington, M. , & Velicer, W. F. (2015). Comparing visual and statistical analysis in single‐case studies using published studies. Multivariate Behavioral Research, 50(2), 162‐183. 10.1080/00273171.2014.973989 PMC4677800 26609876

[jaba923-bib-0041] Harrison, D. A. , & Klein, K. J. (2007). What's the difference? Diversity constructs as separation, variety, or disparity in organizations. Academy of Management Review, 32(4), 1199‐1228. 10.5465/amr.2007.26586096

[jaba923-bib-0042] Hartgerink, C. H. J. , Wicherts, J. M. , & van Assen, M. A. L. M. (2017). Too good to be false: Nonsignificant results revisited. Collabra: Psychology, 3(1), 9. 10.1525/collabra.71

[jaba923-bib-0043] Hartmann, D. P. , & Hall, R. V. (1976). The changing criterion design. Journal of Applied Behavior Analysis, 9(4), 527‐532. 10.1901/jaba.1976.9-527 PMC1312042 1002635

[jaba923-bib-0044] Hayes, S. C. , Hofmann, S. G. , Stanton, C. E. , Carpenter, J. K. , Sanford, B. T. , Curtiss, J. E. , & Ciarrochi, J. (2019). The role of the individual in the coming era of process‐based therapy. Behaviour Research and Therapy, 117(June), 40‐53. 10.1016/j.brat.2018.10.005 30348451

[jaba923-bib-0045] Hedges, L. V. (2019). The statistics of replication. Methodology, 15(1), 3‐14. 10.1027/1614-2241/a000173

[jaba923-bib-0046] Hedges, L. V. , & Schauer, J. M. (2019a). More than one replication study is needed for unambiguous tests of replication. Journal of Educational and Behavioral Statistics, 44(5), 543‐570. 10.3102/1076998619852953

[jaba923-bib-0047] Hedges, L. V. , & Schauer, J. M. (2019b). Statistical analyses for studying replication: Meta‐analytic perspectives. Psychological Methods, 24(5), 557‐570. 10.1037/met0000189 30070547

[jaba923-bib-0048] Hedges, L. V. , & Vevea, J. L. (1998). Fixed‐ and random‐effects models in meta‐analysis. Psychological Methods, 3(4), 486‐504. 10.1037/1082-989X.3.4.486

[jaba923-bib-0049] Heyvaert, M. , & Onghena, P. (2014). Analysis of single‐case data: Randomisation tests for measures of effect size. Neuropsychological Rehabilitation, 24(3‐4), 507‐527. 10.1080/09602011.2013.818564 23865938

[jaba923-bib-0050] Hillary, F. G. , & Medaglia, J. D. (2020). What the replication crisis means for intervention science. International Journal of Psychophysiology, 154(August), 3‐5. 10.1016/j.ijpsycho.2019.05.006 PMC6842660 31082406

[jaba923-bib-0051] Hitchcock, J. H. , Kratochwill, T. R. , & Chezan, L. C. (2015). What Works Clearinghouse standards and generalization of single‐case design evidence. Journal of Behavioral Education, 24(4), 459‐469. 10.1007/s10864-015-9224-1

[jaba923-bib-0052] Horner, R. H. , Carr, E. G. , Halle, J. , McGee, G. , Odom, S. , & Wolery, M. (2005). The use of single‐subject research to identify evidence‐based practice in special education. Exceptional Children, 71(2), 165‐179. 10.1177/001440290507100203

[jaba923-bib-0053] Horner, R. J. , & Odom, S. L. (2014). Constructing single‐case research designs: Logic and options. In T. R. Kratochwill & J. R. Levin (Eds.), Single‐case intervention research: Methodological and statistical advances (pp. 27‐51). American Psychological Association. 10.1037/14376-002

[jaba923-bib-0054] Hyndman, R. J. , & Koehler, A. B. (2006). Another look at measures of forecast accuracy. International Journal of Forecasting, 22(4), 679‐688. 10.1016/j.ijforecast.2006.03.001

[jaba923-bib-0055] Imam, A. A. (2021). Historically recontextualizing Sidman's Tactics: How behavior analysis avoided psychology's methodological Ouroboros. Journal of the Experimental Analysis of Behavior, 115(1), 115‐128. 10.1002/jeab.661 33336404

[jaba923-bib-0056] Iversen, I. H. (2021). Sidman or statistics? Journal of the Experimental Analysis of Behavior, 115(1), 102‐114. 10.1002/jeab.660 33330993

[jaba923-bib-0057] Jacobson, N. S. , & Truax, P. (1991). Clinical significance: A statistical approach to meaningful change in psychotherapy research. Journal of Consulting and Clinical Psychology, 59(1), 12‐19. 10.1037/0022-006X.59.1.12 2002127

[jaba923-bib-0058] Johnson, A. H. , & Cook, B. G. (2019). Preregistration in single‐case design research. Exceptional Children, 86(1), 95‐112. 10.1177/0014402919868529

[jaba923-bib-0059] Karazsia, B. T. (2018). Editorial: New instructions for single‐subject research in the Journal of Pediatric Psychology. Journal of Pediatric Psychology, 43(6), 585‐587. 10.1093/jpepsy/jsy039 29800385

[jaba923-bib-0060] Kazdin, A. E. (1977). Assessing the clinical or applied importance of behavior change through social validation. Behavior Modification, 1(4), 427‐452. 10.1177/014544557714001

[jaba923-bib-0061] Kazdin, A. E. (2020). Single‐case research designs: Methods for clinical and applied settings (3rd ed.). Oxford University Press.

[jaba923-bib-0062] Kazdin, A. E. (2021). Single‐case experimental designs: Characteristics, changes, and challenges. Journal of the Experimental Analysis of Behavior, 115(1), 56‐85. 10.1002/jeab.638 33205436

[jaba923-bib-0063] Kennedy, C. H. (2005). Single‐case designs for educational research. Pearson.

[jaba923-bib-0064] Killeen, P. R. (2005). An alternative to null hypothesis statistical tests. Psychological Science, 16(5), 345‐353. 10.1111/j.0956-7976.2005.01538.x PMC1473027 15869691

[jaba923-bib-0065] Kinney, C. E. L. (2022). A clarification of slope and scale. Behavior Modification, 46(1), 90‐127. 10.1177/0145445520953366 32873062

[jaba923-bib-0066] Kipfmiller, K. J. , Brodhead, M. T. , Wolfe, K. , LaLonde, K. , Sipila, E. S. , Bak, M. S. , & Fisher, M. H. (2019). Training front‐line employees to conduct visual analysis using a clinical decision‐making model. Journal of Behavioral Education, 28(3), 301‐322. 10.1007/s10864-018-09318-1

[jaba923-bib-0067] Kranak, M. P. , Falligant, J. M. , & Hausman, N. L. (2021). Application of automated nonparametric statistical analysis in clinical contexts. Journal of Applied Behavior Analysis, 54(2), 824‐833. 10.1002/jaba.789 33084039

[jaba923-bib-0068] Kratochwill, T. R. , Hitchcock, J. H. , Horner, R. H. , Levin, J. R. , Odom, S. L. , Rindskopf, D. M. , & Shadish, W. R. (2013). Single‐case intervention research design standards. Remedial and Special Education, 34(1), 26‐38. 10.1177/0741932512452794

[jaba923-bib-0069] Kubina, R. M. , Kostewicz, D. E. , Brennan, K. M. , & King, S. A. (2017). A critical review of line graphs in behavior analytic journals. Educational Psychology Review, 29(3), 583‐598. 10.1007/s10648-015-9339-x

[jaba923-bib-0070] L'Abbé, K. A. , Detsky, A. S. , & O'Rourke, K. (1987). Meta‐analysis in clinical research. Annals of Internal Medicine, 107(2), 224‐233. 10.7326/0003-4819-107-2-224 3300460

[jaba923-bib-0071] Lane, J. D. , & Gast, D. L. (2014). Visual analysis in single case experimental design studies: Brief review and guidelines. Neuropsychological Rehabilitation, 24(3‐4), 445‐463. 10.1080/09602011.2013.815636 23883189

[jaba923-bib-0072] Lanovaz, M. J. , & Rapp, J. T. (2016). Using single‐case experiments to support evidence‐based decisions: How much is enough? Behavior Modification, 40(3), 377‐395. 10.1177/0145445515613584 26538276

[jaba923-bib-0073] Lanovaz, M. J. , & Turgeon, S. (2020). How many tiers do we need? Type I errors and power in multiple baseline designs. Perspectives on Behavior Science, 43(3), 605‐616. 10.1007/s40614-020-00263-x PMC7490309 33024931

[jaba923-bib-0074] Lanovaz, M. J. , Turgeon, S. , Cardinal, P. , & Wheatley, T. L. (2019). Using single‐case designs in practical settings: Is within‐subject replication always necessary? Perspectives on Behavior Science, 42(1), 153‐162. 10.1007/s40614-018-0138-9 PMC6701506 31976426

[jaba923-bib-0075] Laraway, S. , Snycerski, S. , Pradhan, S. , & Huitema, B. E. (2019). An overview of scientific reproducibility: Consideration of relevant issues for behavior science/analysis. Perspectives on Behavior Science, 42(1), 33‐57. 10.1007/s40614-019-00193-3 PMC6701706 31976420

[jaba923-bib-0076] Ledford, J. R. , Barton, E. E. , Hardy, J. K. , Elam, K. , Seabolt, J. , Shanks, M. , Hemmeter, M. L. , & Kaiser, A. (2016). What equivocal data from single case comparison studies reveal about evidence‐based practices in early childhood special education. Journal of Early Intervention, 38(2), 79‐91. 10.1177/1053815116648000

[jaba923-bib-0077] Ledford, J. R. , Barton, E. E. , Severini, K. E. , & Zimmerman, K. N. (2019). A primer on single‐case research designs: Contemporary use and analysis. American Journal on Intellectual and Developmental Disabilities, 124(1), 35‐56. 10.1352/1944-7558-124.1.35 30715924

[jaba923-bib-0078] Ledford, J. R. , Barton, E. E. , Severini, K. E. , Zimmerman, K. N. , & Pokorski, E. A. (2019). Visual display of graphic data in single case design studies: Systematic review and expert preference analysis. Education and Training in Autism and Developmental Disabilities, 54(4), 315‐327. https://www.jstor.org/stable/26822511

[jaba923-bib-0079] Ledford, J. R. , & Gast, D. L. (2018). Single case research methodology: Applications in special education and behavioral sciences (3rd ed.). Routledge.

[jaba923-bib-0080] Lehardy, R. K. , Luczynski, K. C. , Hood, S. A. , & McKeown, C. A. (2021). Remote teaching of publication‐quality, single‐case graphs in Microsoft Excel. Journal of Applied Behavior Analysis, 54(3), 1265‐1280. 10.1002/jaba.805 33527372

[jaba923-bib-0081] Levin, J. R. , Ferron, J. M. , & Gafurov, B. S. (2017). Additional comparisons of randomization‐test procedures for single‐case multiple‐baseline designs: Alternative effect types. Journal of School Psychology, 63(August), 13‐34. 10.1016/j.jsp.2017.02.003 28633936

[jaba923-bib-0082] Levin, J. R. , Ferron, J. M. , & Gafurov, B. S. (2021). Investigation of single‐case multiple‐baseline randomization tests of trend and variability. Educational Psychology Review, 33(2), 713–737. 10.1007/s10648-020-09549-7

[jaba923-bib-0083] Maggin, D. M. (2015). Considering generality in the systematic review and meta‐analysis of single‐case research: A response to Hitchcock et al. Journal of Behavioral Education, 24(4), 470‐482. 10.1007/s10864-015-9239-7

[jaba923-bib-0084] Maggin, D. M. , Briesch, A. M. , & Chafouleas, S. M. (2013). An application of the What Works Clearinghouse standards for evaluating single‐subject research: Synthesis of the self‐management literature base. Remedial and Special Education, 34(1), 44‐58. 10.1177/0741932511435176

[jaba923-bib-0085] Maggin, D. M. , Cook, B. G. , & Cook, L. (2018). Using single‐case research designs to examine the effects of interventions in special education. Learning Disabilities Research & Practice, 33(4), 182‐191. 10.1111/ldrp.12184

[jaba923-bib-0086] Maggin, D. M. , Cook, B. G. , & Cook, L. (2019). Making sense of single‐case design effect sizes. Learning Disabilities Research & Practice, 34(3), 124‐132. 10.1111/ldrp.12204

[jaba923-bib-0087] Manolov, R. , Jamieson, M. , Evans, J. J. , & Sierra, V. (2016). A discussion of alternatives for establishing empirical benchmarks for interpreting single‐case effect sizes. Psicológica, 37(2), 209‐234. http://www.uv.es/psicologica/articulos2.16/6Manolov.pdf

[jaba923-bib-0088] Manolov, R. , Moeyaert, M. , & Fingerhut, J. (2022). A priori justification for effect measures in single‐case experimental designs. Perspectives on Behavior Science, 45(1), 156‐189. 10.1007/s40614-021-00282-2 PMC8894525 35342872

[jaba923-bib-0089] Manolov, R. , & Onghena, P. (2018). Analyzing data from single‐case alternating treatments designs. Psychological Methods, 23(3), 480‐504. 10.1037/met0000133 28301199

[jaba923-bib-0090] Manolov, R. , & Tanious, R. (2022). Assessing consistency in single‐case data features using modified Brinley plots. Behavior Modification, 46(3),581‐627. 10.1177/0145445520982969 33371723

[jaba923-bib-0091] Manolov, R. , Tanious, R. , & Fernández‐Castilla, B. (2021). Exploratory graphical analysis of SCED effect sizes at different levels: Sensitivity analysis using modified Brinley plots. 10.31234/osf.io/qfjza

[jaba923-bib-0092] Manolov, R. , Tanious, R. , & Onghena, P. (2022). Quantitative techniques and graphical representations for interpreting results from alternating treatment design. Perspectives on Behavior Science, 45(1), 259‐294. 10.1007/s40614-021-00289-9 PMC8894511 35342876

[jaba923-bib-0093] McDougale, C. B. , Richling, S. M. , Longino, E. B. , & O'Rourke, S. A. (2020). Mastery criteria and maintenance: A descriptive analysis of applied research procedures. Behavior Analysis in Practice, 13(2), 402‐410. 10.1007/s40617-019-00365-2 PMC7314871 32642396

[jaba923-bib-0094] Michiels, B. , Heyvaert, M. , Meulders, A. , & Onghena, P. (2017). Confidence intervals for single‐case effect size measures based on randomization test inversion. Behavior Research Methods, 49(1), 363‐381. 10.3758/s13428-016-0714-4 26927003

[jaba923-bib-0095] Miller, M. J. (1985). Analyzing client change graphically. Journal of Counseling and Development, 63(8), 491‐494. 10.1002/j.1556-6676.1985.tb02743.x

[jaba923-bib-0096] Mitteer, D. R. , Greer, B. D. , Fisher, W. W. , & Cohrs, V. L. (2018). Teaching behavior technicians to create publication‐quality, single‐case design graphs in GraphPad Prism 7. Journal of Applied Behavior Analysis, 51(4), 998‐1010. 10.1002/jaba.483 PMC6188791 29971776

[jaba923-bib-0097] Moeyaert, M. , Yang, P. , & Xu, X. , & Kim, E. (2021). Characteristics of moderators in meta‐analyses of single‐case experimental design studies. Behavior Modification. Advance online publication. 10.1177/01454455211002111 33759586

[jaba923-bib-0098] Morley, S. (2018). Single‐case methods in clinical psychology: A practical guide. Routledge.

[jaba923-bib-0099] Natesan, P. (2019). Fitting Bayesian models for single‐case experimental designs: A tutorial. Methodology, 15(4), 147‐156. 10.1027/1614-2241/a000180

[jaba923-bib-0100] Nikles, J. , Daza, E. J. , McDonald, S. , Hekler, E. , & Schork, N. (2021). Editorial: Creating evidence from real world patient digital data. Frontiers in Computer Science, 61. 10.3389/fcomp.2020.636996

[jaba923-bib-0101] Ninci, J. (2019). Single‐case data analysis: A practitioner guide for accurate and reliable decisions. Behavior Modification. 10.1177/0145445519867054 31441315

[jaba923-bib-0102] Ninci, J. , Vannest, K. J. , Willson, V. , & Zhang, N. (2015). Interrater agreement between visual analysts of single‐case data: A meta‐analysis. Behavior Modification, 39(4), 510‐541. 10.1177/0145445515581327 25878161

[jaba923-bib-0103] Normand, M. P. (2016). Less is more: Psychologists can learn more by studying fewer people. Frontiers in Psychology, 7, e934. 10.3389/fpsyg.2016.00934 PMC4911349 27379004

[jaba923-bib-0104] Onghena, P. , & Edgington, E. S. (2005). Customization of pain treatments: Single‐case design and analysis. Clinical Journal of Pain, 21(1), 56‐68. 10.1097/00002508-200501000-00007 15599132

[jaba923-bib-0105] Parker, R. I. , Cryer, J. , & Byrns, G. (2006). Controlling baseline trend in single‐case research. School Psychology Quarterly, 21(4), 418‐443. 10.1037/h0084131

[jaba923-bib-0106] Parker, R. I. , & Vannest, K. J. (2009). An improved effect size for single‐case research: Nonoverlap of all pairs. Behavior Therapy, 40(4), 357‐367. 10.1016/j.beth.2008.10.006 19892081

[jaba923-bib-0107] Parker, R. I. , & Vannest, K. J. (2012). Bottom‐up analysis of single‐case research designs. Journal of Behavioral Education, 21(3), 254‐265. 10.1007/s10864-012-9153-1

[jaba923-bib-0108] Parker, R. I. , Vannest, K. J. , & Davis, J. L. (2011). Effect size in single‐case research: A review of nine nonoverlap techniques. Behavior Modification, 35(4), 303‐322. 10.1177/0145445511399147 21411481

[jaba923-bib-0109] Peltier, C. , McKenna, J. W. , Sinclair, T. E. , Garwood, J. , & Vannest, K. J. (2022). Brief report: Ordinate scaling and axis proportions of single‐case graphs in two prominent EBD journals from 2010 to 2019. Behavioral Disorders, 47(2), 134‐148. 10.1177/0198742920982587

[jaba923-bib-0110] Peltier, C. , Morano, S. , Shin, M. , Stevenson, N. , & McKenna, J. W. (2021). A decade review of single‐case graph construction in the field of learning disabilities. Learning Disabilities Research & Practice, 36(2), 121‐135. 10.1111/ldrp.12245

[jaba923-bib-0111] Peltier, C. , Muharib, R. , Haas, A. , & Dowdy, A. (2022). A decade review of two potential analysis altering variables in graph construction. Journal of Autism and Developmental Disorders, 52(2), 714–724. 10.1007/s10803-021-04959-0 33763782

[jaba923-bib-0112] Perone, M. (1999). Statistical inference in behavior analysis: Experimental control is better. The Behavior Analyst, 22(2), 109‐116. 10.1007/BF03391988 PMC2731354 22478328

[jaba923-bib-0113] Pfadt, A. , & Wheeler, D. J. (1995). Using statistical process control to make data‐based clinical decisions. Journal of Applied Behavior Analysis, 28(3), 349‐370. 10.1901/jaba.1995.28-349 PMC1279837 7592154

[jaba923-bib-0114] Porcino, A. J. , Shamseer, L. , Chan, A. W. , Kravitz, R. L. , Orkin, A. , Punja, S. , Ravaud, P. , Schmid, C. H. , & Vohra, S. (2020). SPIRIT extension and elaboration for n‐of‐1 trials: SPENT 2019 checklist. BMJ, 368, m122. 10.1136/bmj.m122 32107202

[jaba923-bib-0115] Radley, K. C. , Dart, E. H. , & Wright, S. J. (2018). The effect of data points per x‐ to y‐axis ratio on visual analysts' evaluation of single‐case graphs. School Psychology Quarterly, 33(2), 314‐322. 10.1037/spq0000243 29446963

[jaba923-bib-0116] Riley‐Tillman, T. C. , Burns, M. K. , & Kilgus, S. P. (2020). Evaluating educational interventions: Single‐case design for measuring response to intervention (2nd ed.). The Guilford Press.

[jaba923-bib-0117] Roane, H. S. , Fisher, W. W. , Kelley, M. E. , Mevers, J. L. , & Bouxsein, K. J. (2013). Using modified visual‐inspection criteria to interpret functional analysis outcomes. Journal of Applied Behavior Analysis, 46(1), 130‐146. 10.1002/jaba.13 24114090

[jaba923-bib-0118] Sanabria, F. , & Killeen, P. R. (2007). Better statistics for better decisions: Rejecting null hypothesis statistical tests in favor of replication statistics. Psychology in the Schools, 44(5), 471‐481. 10.1002/pits.20239 PMC2613360 19122766

[jaba923-bib-0119] Schauer, J. M. , Fitzgerald, K. G. , Peko‐Spicer, S. , Whalen, M. C. , Zejnullahi, R. , & Hedges, L. V. (2021). An evaluation of statistical methods for aggregate patterns of replication failure. The Annals of Applied Statistics, 15(1), 208‐229. 10.1214/20-AOAS1387

[jaba923-bib-0120] Shepley, C. , Ault, M. J. , Ortiz, K. , Vogler, J. C. , & McGee, M. (2020). An exploratory analysis of quality indicators in adapted alternating treatments designs. Topics in Early Childhood Special Education, 39(4), 226‐237. 10.1177/0271121418820429

[jaba923-bib-0121] Sidman, M. (1960). Tactics of scientific research. Basic Books.

[jaba923-bib-0122] Skinner, C. H. , McClurg, V. , Crewdson, M. , Coleman, M. B. , Bennett, J. , Fowler, K. , & Killion, J. B. (2021). Alternating treatments designs: Interpretation challenges and design solutions for validating and comparing interventions. Psychology in the Schools, 59(4), 678‐697. 10.1002/pits.2263820

[jaba923-bib-0123] Snodgrass, M. R. , Chung, M. Y. , Meadan, H. , & Halle, J. W. (2018). Social validity in single‐case research: A systematic literature review of prevalence and application. Research in Developmental Disabilities, 74(March), 160‐173. 10.1016/j.ridd.2018.01.007 29413430

[jaba923-bib-0124] Snodgrass, M. R. , Meadan, H. , Chung, M. Y. , & Biggs, E. E. (2022). Graphing the intersection of rate and fidelity in single‐case research. Behavior Analysis in Practice, 15(1), 284‐294. 10.1007/s40617-021-00556-w PMC8854548 35340379

[jaba923-bib-0125] Spear, C. F. , Strickland‐Cohen, M. K. , Romer, N. , & Albin, R. W. (2013). An examination of social validity within single‐case research with students with emotional and behavioral disorders. Remedial and Special Education, 34(6), 357‐370. 10.1177/0741932513490809

[jaba923-bib-0126] Tanious, R. , De, T. K. , Michiels, B. , Van den Noortgate, W. , & Onghena, P. (2020). Assessing consistency in single‐case A‐B‐A‐B phase designs. Behavior Modification, 44(4), 518‐551. 10.1177/0145445519837726 30931585

[jaba923-bib-0127] Tanious, R. , & Onghena, P. (2021). A systematic review of applied single‐case research published between 2016 and 2018: Study designs, randomization, data aspects, and data analysis. Behavior Research Methods, 53(4), 1371‐1384. 10.3758/s13428-020-01502-4 33104956

[jaba923-bib-0128] Tarlow, K. R. , Brossart, D. F. , McCammon, A. M. , Giovanetti, A. J. , Belle, M. C. , & Philip, J. (2021). Reliable visual analysis of single‐case data: A comparison of rating, ranking, and pairwise methods. Cogent Psychology, 8(1), 1911076. 10.1080/23311908.2021.1911076

[jaba923-bib-0129] Tate, R. L. , & Perdices, M. (2019). Single‐case experimental designs for clinical research and neurorehabilitation settings: Planning, conduct, analysis, and reporting. Routledge.

[jaba923-bib-0130] Tate, R. L. , Perdices, M. , Rosenkoetter, U. , Wakim, D. , Godbee, K. , Togher, L. , & McDonald, S. (2013). Revision of a method quality rating scale for single‐case experimental designs and n‐of‐1 trials: The 15‐item Risk of Bias in N‐of‐1 Trials (RoBiNT) Scale. Neuropsychological Rehabilitation, 23(5), 619‐638. 10.1080/09602011.2013.824383 24050810

[jaba923-bib-0131] Thirumanickam, A. , Raghavendra, P. , McMillan, J. M. , & van Steenbrugge, W. (2018). Effectiveness of video‐based modelling to facilitate conversational turn taking of adolescents with autism spectrum disorder who use AAC. AAC: Augmentative and Alternative Communication, 34(4), 311‐322. 10.1080/07434618.2018.1523948 30456987

[jaba923-bib-0132] Tincani, M. , & Travers, J. (2018). Publishing single‐case research design studies that do not demonstrate experimental control. Remedial and Special Education, 39(2), 118‐128. 10.1177/0741932517697447

[jaba923-bib-0133] Tincani, M. , & Travers, J. (2019). Replication research, publication bias, and applied behavior analysis. Perspectives on Behavior Science, 42(1), 59‐75. 10.1007/s40614-019-00191-5 PMC6701502 31976421

[jaba923-bib-0134] U. S. Department of Education (2020). What Works Clearinghouse Standards Handbook . https://ies.ed.gov/ncee/wwc/handbooks

[jaba923-bib-0135] Vannest, K. J. , & Sallese, M. R. (2021). Benchmarking effect sizes in single‐case experimental designs. Evidence‐Based Communication Assessment and Intervention, 15(3), 142‐165. 10.1080/17489539.2021.1886412

[jaba923-bib-0136] Walker, S. G. , & Carr, J. E. (2021). Generality of findings from single‐case designs: It's not all about the “n.” Behavior Analysis in Practice, 14(4), 991‐995. 10.1007/s40617-020-00547-3 PMC8586328 34868812

[jaba923-bib-0137] Wolfe, K. , Barton, E. E. , & Meadan, H. (2019). Systematic protocols for the visual analysis of single‐case research data. Behavior Analysis in Practice, 12(2), 491‐502. 10.1007/s40617-019-00336-7 PMC6745757 31976257

[jaba923-bib-0138] Wolfe, K. , & McCammon, M. N. (2022). The analysis of single‐case research data: Current instructional practices. Journal of Behavioral Education, 31(1), 28‐42. 10.1007/s10864-020-09403-4

[jaba923-bib-0139] Wolfe, K. , McCammon, M. N. , LeJeune, L. M. , & Holt, A. K. (2021). Training preservice practitioners to make data‐based instructional decisions. Journal of Behavioral Education. 10.1007/s10864-021-09439-0

